# Deficiency of *TET3* leads to a genome-wide DNA hypermethylation episignature in human whole blood

**DOI:** 10.1038/s41525-021-00256-y

**Published:** 2021-11-08

**Authors:** Michael A. Levy, David B. Beck, Kay Metcalfe, Sofia Douzgou, Sivagamy Sithambaram, Trudie Cottrell, Muhammad Ansar, Jennifer Kerkhof, Cyril Mignot, Marie-Christine Nougues, Boris Keren, Hannah W. Moore, Renske Oegema, Jacques C. Giltay, Marleen Simon, Richard H. van Jaarsveld, Jessica Bos, Mieke van Haelst, M. Mahdi Motazacker, Elles M. J. Boon, Gijs W. E. Santen, Claudia A. L. Ruivenkamp, Marielle Alders, Teresa Romeo Luperchio, Leandros Boukas, Keri Ramsey, Vinodh Narayanan, G. Bradley Schaefer, Roberto Bonasio, Kimberly F. Doheny, Roger E. Stevenson, Siddharth Banka, Bekim Sadikovic, Jill A. Fahrner

**Affiliations:** 1 Molecular Genetics Laboratory, Molecular Diagnostics Division, London Health Sciences Centre, London, ON N6A5W9 Canada; 2 National Human Genome Research Institute, Bethesda, MD 20892 USA; 3 Division of Evolution, Infection and Genomics, School of Biological Sciences, Faculty of Biology, Medicine and Health, University of Manchester, Manchester, M13 9PL UK; 4 Manchester Centre for Genomic Medicine, St Mary’s Hospital, Health Innovation Manchester, Manchester University NHS Foundation Trust, Manchester, M13 9WL UK; 5 Department of Biochemistry, Faculty of Biological Sciences, Quaid-I-Azam University, 45320 Islamabad, Pakistan; 6 Assistance Publique-Hopitaux de Paris, Sorbonne Université, Departement de Génétique, Groupe Hospitalier Pitie-Salpetriere et Hopital Trousseau, Paris, 75651 France; 7 Department of Neuropediatrics, Armand Trousseau Hospital, Assistance Publique-Hopitaux de Paris, Paris, 75012 France; 8 Laboratoire de génétique, Hôpital Pïtié-Salpêtrière, Assistance Publique-Hopitaux de Paris, Paris, 75013 France; 9 Greenwood Genetic Center, Greenwood, SC 29646 USA; 10 Department of Genetics, University Medical Center Utrecht, Utrecht University, Utrecht, The Netherlands; 11 Section Clinical Genetics, Department Human Genetics, Amsterdam University Medical Centers, Amsterdam, The Netherlands; 12 Department of Human Genetics, Laboratory of Genome Diagnostics, Amsterdam UMC, University of Amsterdam, Meibergdreef 9, Amsterdam, Netherlands; 13 Department of Human Genetics, VU University Medical Center Amsterdam, Amsterdam UMC, van der Boechorststraat 7, 1081 BT Amsterdam, The Netherlands; 14 Department of Clinical Genetics, Leiden University Medical Center, Leiden, The Netherlands; 15 Department of Human Genetics, Amsterdam Reproduction & Development Research Institute, Amsterdam UMC, University of Amsterdam, Meibergdreef 9, Amsterdam, The Netherlands; 16 Department of Genetic Medicine, Johns Hopkins University School of Medicine, Baltimore, MD 21205 USA; 17 Department of Biostatistics, Johns Hopkins University, Baltimore, MD 21205 USA; 18 Center for Rare Childhood Disorders, Translational Genomics Research Institute, Phoenix, AZ USA; 19 University of Arkansas for Medical Sciences, Springdale, AR 72762 USA; 20 Department of Cell and Developmental Biology, University of Pennsylvania Perelman School of Medicine, Philadelphia, PA 19104 USA; 21 Epigenetics Institute, University of Pennsylvania Perelman School of Medicine, Philadelphia, PA 19104 USA; 22 Center for Inherited Disease Research, Johns Hopkins University School of Medicine, Baltimore, MD USA; 23 Department of Pathology and Laboratory Medicine, Western University, London, ON N6A5W9 Canada; 24 Department of Pediatrics, Johns Hopkins University School of Medicine, Baltimore, MD 21205 USA

**Keywords:** Epigenomics, DNA methylation, Neurodevelopmental disorders, Diagnostic markers, Neurodevelopmental disorders

## Abstract

*TET3* encodes an essential dioxygenase involved in epigenetic regulation through DNA demethylation. *TET3* deficiency, or Beck-Fahrner syndrome (BEFAHRS; MIM: 618798), is a recently described neurodevelopmental disorder of the DNA demethylation machinery with a nonspecific phenotype resembling other chromatin-modifying disorders, but inconsistent variant types and inheritance patterns pose diagnostic challenges. Given TET3’s direct role in regulating 5-methylcytosine and recent identification of syndrome-specific DNA methylation profiles, we analyzed genome-wide DNA methylation in whole blood of *TET3-*deficient individuals and identified an episignature that distinguishes affected and unaffected individuals and those with mono-allelic and bi-allelic pathogenic variants. Validation and testing of the episignature correctly categorized known *TET3* variants and determined pathogenicity of variants of uncertain significance. Clinical utility was demonstrated when the episignature alone identified an affected individual from over 1000 undiagnosed cases and was confirmed upon distinguishing *TET3-*deficient individuals from those with 46 other disorders. The *TET3*-deficient signature - and the signature resulting from activating mutations in *DNMT1* which normally opposes TET3 - are characterized by hypermethylation, which for BEFAHRS involves CpG sites that may be biologically relevant. This work expands the role of epi-phenotyping in molecular diagnosis and reveals genome-wide DNA methylation profiling as a quantitative, functional readout for characterization of this new biochemical category of disease.

## Introduction

Mendelian disorders of the epigenetic machinery, otherwise known as chromatin modifying disorders, are a rapidly growing group of congenital disorders resulting from germ-line mutations in genes encoding components of the epigenetic machinery^1–3^. The epigenetic and chromatin modifying machinery consists of enzymes, including chromatin remodelers and writers and erasers of epigenetic marks, as well as non-enzymatic readers of these marks, and genetic disruption in any of these components can have broad genome-wide epigenetic consequences^4^. The two main types of epigenetic marks are histone post-translational modifications and DNA methylation (also referred to as 5-methylcytosine; 5mC), and their collective role is to dynamically regulate temporal and cell type-specific gene expression^5–7^. Each of these broad groups has its own set of writers, erasers, and readers of epigenetic marks, and the vast majority of these disorders result from mutations in genes encoding components of the histone modification system with far fewer impacting the DNA methylation machinery^1,4^. Whereas disorders involving writers and readers of DNA methylation have been known for some time, only recently was the first neurodevelopmental disorder impacting the DNA methylation eraser system, *TET3* deficiency, or Beck-Fahrner syndrome (BEFAHRS; MIM: 618798), delineated^8,9^.

Like other Mendelian disorders of the epigenetic machinery, BEFAHRS is characterized by intellectual disability (ID) and other neurobehavioral manifestations, including hypotonia, autism, and epilepsy, as well as growth abnormalities^8^. Distinct from most other disorders within this group, the inheritance pattern of BEFAHRS is mixed and includes autosomal recessive and autosomal dominant forms. Pathogenic missense variants can be mono-allelic or bi-allelic, occur within the catalytic domain at highly conserved residues, and most result in reduced but not absent enzymatic activity in vitro^8^. Pathogenic frameshift variants occur throughout the coding region and have only been described in mono-allelic form, raising the possibility of haploinsufficiency in some cases. These observations suggest that reduced enzyme activity may be a unifying disease mechanism irrespective of inheritance pattern and that at least some residual TET3 activity remains and is required for viability^8^. The identification of a frameshift and a nonsense variant in the last exon could suggest an additional (likely dominant-negative) disease mechanism. Therefore, whereas BEFAHRS is clearly a distinct disease entity, the above observations suggest considerable variability with regard to inheritance patterns, variant types, and potential mutation mechanisms, as well as phenotypic features^8^. Moreover, the latter are non-specific and overlap significantly with other Mendelian disorders of the epigenetic machinery^1–4^. The high variability and non-specific clinical features of BEFAHRS have the potential to lead to challenges in diagnosis and in understanding the molecular basis of disease.

Recent diagnostic advances, which also have the potential to shed light on disease pathogenesis, have come from genome-wide DNA methylation profiling of DNA isolated from whole blood of patients^10,11^. Sensitive and specific genome-wide DNA methylation patterns, often referred to as “episignatures,” have been reported in multiple Mendelian disorders of the epigenetic machinery and related neurodevelopmental and multiple congenital anomaly syndromes^10–19^. DNA methylation arrays exhibit diagnostic utility in individuals with unknown conditions and are now being offered as a clinical diagnostic test for a subset of these conditions^10,20^. Disorders include those impacting histones and the histone modification system^12,16,18,19,21^, the DNA methylation system^13^, and the chromatin remodeler system^14,15,17^. Episignatures can differentiate highly related disorders from one another, for example Weaver syndrome (MIM: 277590) from multiple related overgrowth syndromes^18^ and Kabuki syndrome 1 (KS1; MIM: 147920) from CHARGE syndrome (MIM: 214800)^22^. Moreover, two distinct and specific DNA methylation signatures, which correlate with gene variant position, have been described for *KMT2D-*related disorders—one for classic KS1 resulting from mutations throughout the coding region and one for a newly reported disorder resulting from variants localized to exons 38 and 39^23^—as well as for the chromatin-modifying disorder, Helsmoortel-van der Aa syndrome (MIM: 615873)^24^.

Because of these recent reports identifying highly sensitive and specific genome-wide DNA methylation signatures in blood associated with a growing number of Mendelian disorders of the epigenetic machinery, and because BEFAHRS directly impacts the DNA demethylation machinery, we performed genome-wide DNA methylation profiling on a subset of affected individuals from our previously reported cohort^8^, their parents and other family members, additional individuals with presumed pathogenic variants or variants of uncertain significance (VUS’s) in *TET3*, and additional unrelated age-matched and sex-matched controls. We identified a genome-wide DNA methylation signature that differentiates *TET3*-deficient individuals from unaffected controls and from individuals with 46 distinct neurodevelopmental and multiple congenital anomaly syndromes. Similar to the *DNMT1* episignature previously identified in a family with Autosomal Dominant Cerebellar Ataxia Deafness and Narcolepsy (ADCADN) syndrome^13^, the *TET3* episignature is characterized by overall hypermethylation at individual CpG sites. Moreover, the most differentially-methylated clusters of CpGs are associated with protein-coding genes that are highly expressed in fetal neurons and may be phenotypically-relevant. The *TET3* episignature has the ability to distinguish between individuals with mono-allelic and bi-allelic *TET3* variants. After identifying the DNA methylation signature using a discovery cohort, we confirmed the newly generated episignature on a distinct validation cohort. Subsequent testing of a refined signature was able to clarify the affected status of additional probands with VUS’s and identify a *TET3*-deficient individual without prior knowledge of the genetic mutation. Additionally, we further expand and refine the clinical spectrum of BEFAHRS by describing the genotypes and phenotypes of eight individuals from five families with deficiency of *TET3* not previously reported. Together our results provide a better understanding of the spectrum of BEFAHRS and highlight the utility of DNA methylation analysis to aid in genetic diagnosis and in the characterization of new syndromes.

## Results

### *TET3*-deficient peripheral blood samples show an overall increase in genome-wide DNA methylation

**Fig. 1:**
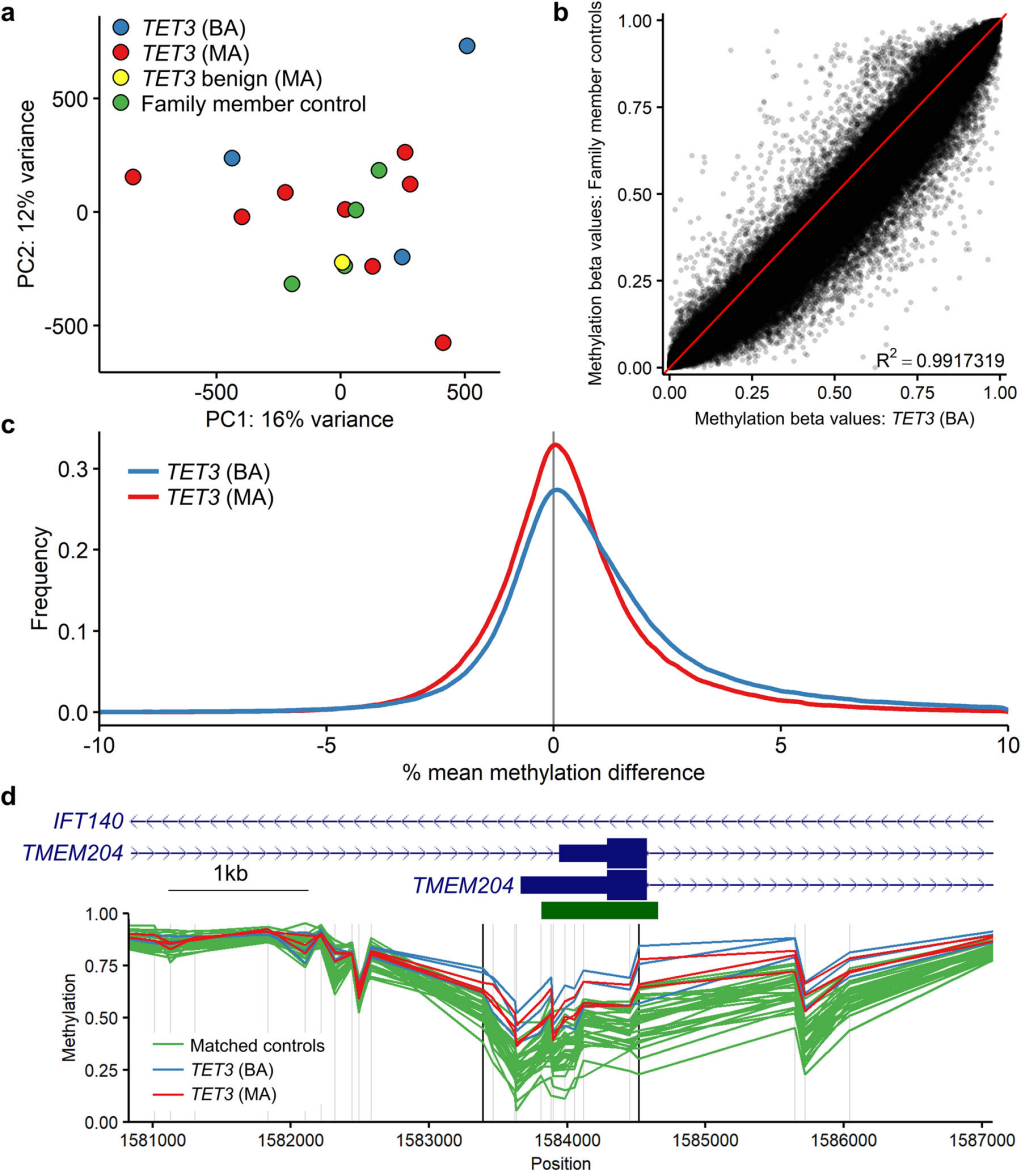
Overall genome-wide DNA methylation in the *TET3* cohort samples. **a** Principal components analysis of the *TET3* discovery and validation cohorts (samples 1–16, Table 1; *n* = 3 *TET3* (BA), blue; *n* = 8 *TET3* (MA), red; *n* = 1 *TET3* benign (MA), yellow; and *n* = 4 family member controls, green). **b** Comparison of mean methylation between the *TET3* (BA) samples (*n* = 3) and family member controls (*n* = 4). *R*^2^ value calculated using Pearson’s correlation coefficient. **c** Difference in mean methylation between the *TET3* (BA) samples (*n* = 3) and family member controls (*n* = 4) and the *TET3* (MA) samples (*n* = 3) and family member controls (*n* = 4). A Kolmogorov-Smirnov test was used to compare the two non-normal distributions and showed the difference to be statistically significant; *D* = 0.02, *P* < 2.2e−16. **d** Differentially methylated region (DMR) at the transcription start site of *TMEM204*, which overlaps with an intron of *IFT140*. DNA methylation is compared between *TET3* (BA) samples (*n* = 3), *TET3* (MA) samples (*n* = 3), and matched controls (*n* = 30). The horizontal green bar indicates a CpG island. Vertical gray lines indicate the location of microarray CpG probes, vertical black lines indicate the boundary of the identified DMR. *TET3* (BA), samples with bi-allelic pathogenic *TET3* variants; *TET3* (MA), samples with mono-allelic pathogenic *TET3* variants; *TET3* benign (MA), sample with mono-allelic *TET3* variant that did not reduce catalytic activity in vitro^8^*;* family member controls, family members of affected individuals lacking *TET3* variants; matched controls, age-and sex-matched controls.

Given the role of TET3 in DNA demethylation, we sought to determine whether loss of functional *TET3* would cause a detectable genome-wide increase in DNA methylation. Principal component analysis (PCA) showed that samples from individuals with pathogenic *TET3* variants, benign *TET3* variants, and family member control individuals lacking *TET3* variants were interspersed, with no particular group showing a distinct cluster (Fig. 1a). This indicates that there are no large differences in DNA methylation between sample types.

We next compared methylation beta values between the samples with bi-allelic and mono-allelic pathogenic *TET3* variants and the four *TET3* cohort controls. Beta values are estimates of methylation based on the ratio of the intensities of methylated to total methylated plus unmethylated probes. Median methylation values for the controls, mono-allelic, and bi-allelic samples were 0.787, 0.789, and 0.801, respectively. Mean methylation values for the same sample groups were 0.591, 0.595, and 0.603. DNA methylation between the bi-allelic *TET3* samples and controls was highly correlated (*r*^2^ = 0.9917319); however, the scatter plot trended toward higher methylation in the samples with pathogenic *TET3* variants (Fig. 1b). We then calculated the difference between the mono-allelic and control samples and the bi-allelic and control samples and plotted the distribution of differences. The *TET3-*deficient samples were slightly skewed towards increased methylation, with the bi-allelic samples showing a stronger skew compared to mono-allelic samples (Fig. 1c).

We next looked for clusters of differentially methylated CpGs to identify differentially methylated regions (DMRs). We compared the six known pathogenic *TET3* samples (three with bi-allelic variants and three with mono-allelic variants) to a set of 30 unrelated age- and sex-matched controls (five control samples for every *TET3*-deficient sample). This identified 50 DMRs, all of which had an increase in methylation in the *TET3-*deficient samples. The full list of DMRs is provided (Supplementary Data 1). The most statistically significant DMR was located at the transcription start site (TSS) of *TMEM204* (Fig. 1d), which is highly expressed in neurons^25^ and contains a CpG island that is occasionally hypermethylated in cancer^26^. The *TMEM204* transcription unit overlaps with *IFT140*, which is on the opposite (antisense) strand (Fig. 1d), is also highly expressed in brain, and is a disease gene known to cause retinitis pigmentosa (MIM: 617781) or Short-rib thoracic dysplasia 9 (MIM: 266920) with associated growth abnormalities and neurologic deficits in some cases (Supplementary Data 1). Overall, 35 of the 39 (90%) protein-coding transcripts associated with these DMRs were expressed in brain according to the GTEx track in the UCSC browser (https://genome.ucsc.edu). We therefore looked more closely at expression of these genes in disease-relevant cell types, namely human fetal cerebral excitatory and inhibitory neurons^27^, and found that in these two cell types, the genes associated with the 50 DMRs are expressed at significantly higher levels than other genes, and this is more pronounced in excitatory neurons (Supplementary Fig. 1). Twenty of the associated genes encoded proteins whose function, if disrupted, would be predicted to lead to one or more phenotypic features of BEFAHRs (Supplementary Data 1). Fourteen of the DMRs fell within the most telomeric band of their respective chromosome, thirteen had associated lncRNAs, and eight were associated with loci that had overlapping transcripts (Supplementary Data 1). Together, the above results indicate that there is a small but detectable increase in DNA methylation in *TET3*-deficient samples across the genome with larger increases found in specific regions (DMRs), some of which may be biologically relevant.

### *TET3*-deficient samples generate a predominantly hypermethylated DNA episignature

**Fig. 2:**
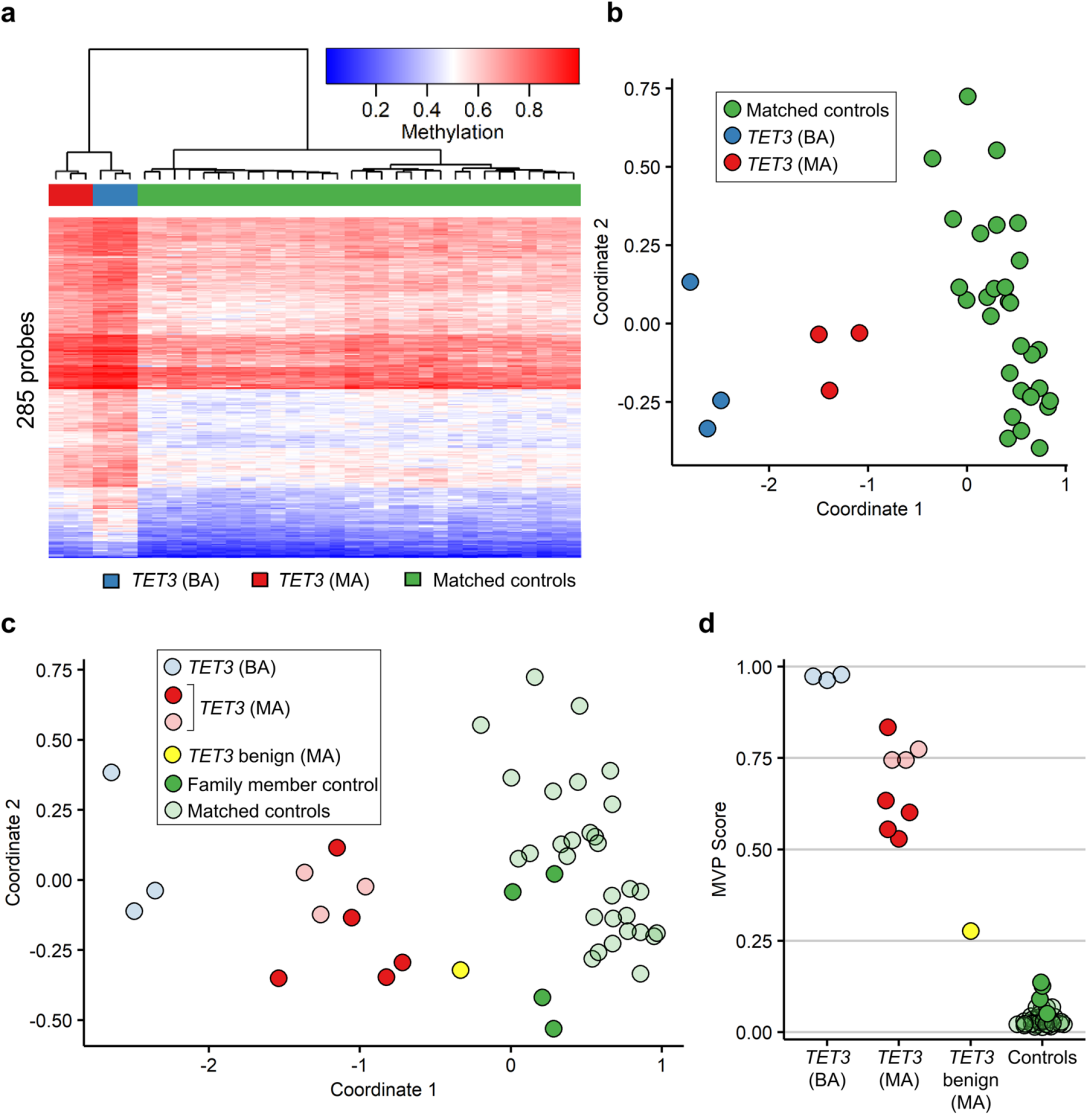
Identification and validation of an initial *TET3* episignature. **a** Hierarchical clustering of the *TET3* signature discovery samples (*n* = 3 *TET3* (BA), blue; *n* = 3 *TET3* (MA), red) and matched controls (*n* = 30, green) after the initial round of episignature discovery. Each row of the heatmap represents one CpG probe, and each column represents one individual’s sample. The heatmap color scale from blue to red represents the DNA methylation level (beta value) from 0 (no methylation) to 1 (fully methylated). **b** Multi-dimensional scaling (MDS) plot of the same signature discovery samples. MDS was performed by scaling of the pair-wise Euclidean distances between samples. **c** MDS plot and **d** methylation variant pathogenicity (MVP) score plot of the signature discovery and validation samples. The signature discovery samples are shown partially transparent and were used for training (*n* = 3 *TET3* (BA), light blue; *n* = 3 *TET3* (MA), light red; *n* = 30 matched controls, light green), and the signature validation samples are opaque and were used for testing (*n* = 5 *TET3* (MA), bright red; *n* = 1 *TET3* benign (MA), bright yellow; *n* = 4 family member controls, bright green). *TET3* (BA), samples with bi-allelic pathogenic *TET3* variants; *TET3* (MA), samples with mono-allelic pathogenic *TET3* variants; *TET3* benign (MA), sample with *TET3* variant that did not reduce catalytic activity in vitro^8^; family member controls, family members of affected individuals lacking *TET3* variants; matched controls, age-matched and sex-matched controls. See Table 1 for description of samples.

While DMRs contain clusters of differentially methylated CpGs, they do not necessarily contain the individual differentially methylated CpGs with the most significant changes—for example, the lowest p values or highest methylation differences—across the genome. We next sought to identify a set of individual CpG probes which could reliably differentiate samples with pathogenic variants in *TET3* from control samples. Using six *TET3-*deficient samples—three with bi-allelic *TET3* variants and three with mono-allelic *TET3* variants (signature discovery samples 1–6, Table 1)—and a set of 30 unrelated age-matched and sex-matched controls (five control samples for every *TET3*-deficient sample), we identified 5315 probes with a mean methylation difference of at least 10% between the *TET3-*deficient and control samples, 1527 probes with an adjusted *p* value <0.001, and 344 probes fulfilling both criteria. After receiver operating curve (ROC) analysis and correlation filtering we obtained a final list of 285 probes, (283 of which had increased DNA methylation), comprising DNA methylation signature 1. Hierarchical clustering clearly separated the *TET3-*deficient and control samples, with the samples with bi-allelic *TET3* variants showing a more robust signature (a higher increase in DNA methylation) than the samples with mono-allelic *TET3* variants (Fig. 2a). Multidimensional scaling (MDS) clustered the samples into three groups, with the mono-allelic samples localizing between bi-allelic samples and controls (Fig. 2b).

**Table 1:** Sample list and variants.

Sample	Patient sex	Patient age (years)	Batch	Used for	Predicted pathogenicity	Sample type	*TET3* variant(s)
1^a^	Female	3	1	Signature discovery	Pathogenic	*TET3* (BA)	c.3215T>G (p.Phe1072Cys)^c^; c.3226G>A (p.Ala1076Thr)^c^
2^a^	Male	21	1	Signature discovery	Pathogenic	*TET3* (BA)	c.2722G>T (p.Val908Leu); c.2722G>T (p.Val908Leu)^c^
3^a^	Female	27	1	Signature discovery	Pathogenic	*TET3* (BA)	c.2722G>T (p.Val908Leu); c.2722G>T (p.Val908Leu)^c^
4^a^	Male	5	1	Signature discovery	Pathogenic	*TET3* (MA)	c.4977_4983del (p.His1660Profs*52)
5^a^	Male	57	1	Signature discovery	Pathogenic	*TET3* (MA)	c.4977_4983del (p.His1660Profs*52)
6^a^	Female	11	1	Signature discovery	Pathogenic	*TET3* (MA^b^)	c.3265G>A (p.Val1089Met)^c^; c.2254C>T (p.Arg752Cys)^c^
7	Female	10	1	Signature validation	NA	Family member control^d^	Familial variant absent by Sanger sequencing
8	Male	23	1	Signature validation	NA	Family member control^e^	Familial variant absent by Sanger sequencing
9	Female	44	1	Signature validation	NA	Family member control^f^	Familial variant absent by exome sequencing
10	Female	10	1	Signature validation	NA	Family member control^g^	Familial variants absent by Sanger, exome sequencing
11	Male	50	1	Signature validation	Benign	*TET3* (MA)	c.2254C>T (p.Arg752Cys)^c^
12^a^	Male	6	2	Signature validation	Pathogenic	*TET3* (MA)	c.5083C>T (p.Gln1695*)
13^a^	Male	5	2	Signature validation	Pathogenic	*TET3* (MA)	c.3100C>T (p.Arg1034*)
14^a^	Female	46	1	Signature validation	Pathogenic	*TET3* (MA)	c.3265G>A (p.Val1089Met)^c^
15^a^	Male	64	1	Signature validation	Pathogenic	*TET3* (MA)	c.2722G>T (p.Val908Leu)^c^
16^a^	Female	28	1	Signature validation	Pathogenic	*TET3* (MA)	c.3226G>A (p.Ala1076Thr)^c^
17	Female	2	2	Testing	Unknown	*TET3* VUS (BA)	c.1483C>T (p.Pro495Ser); c.3883G>A (p.Val1295Ile)
18	Female	11	1	Testing	Unknown	*TET3* VUS (BA)	c.4513G>A (p.Gly1505Arg); c. 5237G>C (p.Trp1746Ser)
19	Female	27	2	Testing	Unknown	*TET3* VUS (MA)	c.1483C>T (p.Pro495Ser)
20	Male	46	2	Testing	Unknown	*TET3* VUS (MA)	c.3883G>A (p.Val1295Ile)
21^a^	Male	21	2	Testing	Unknown	*TET3* VUS (MA)	c.2732G>A (p.Arg911Gln)
22^a^	Male	9	4	Testing	Unknown	*TET3* VUS (MA)	c.5048G>A p.(Arg1683His)
23	Female	42	1	Testing	Unknown	*TET3* VUS (MA)	c.4513G>A (p.Gly1505Arg)
24	Male	47	1	Testing	Unknown	*TET3* VUS (MA)	c.5237G>C (p.Trp1746Ser)
25^a^	Female	54	1	Testing	Unknown	*TET3* VUS (MA)	c.2036dupC (p.Thr680Tyrfs*26)^h^
26^a^	Male	6	1	Testing	Unknown	*TET3* VUS (MA)	c.2036dupC (p.Thr680Tyrfs*26)^h^
27^a^	Male	1	3	Testing	Unknown	Episign screen	c.738C>A (p.Cys246*)

*BA* bi-allelic, *MA* mono-allelic, *NA* not applicable.

^a^*TET3*-deficient pathogenic samples used to identify the final DNA methylation episignature.

^b^Considered mono-allelic because only the c.3265G>A (p.Val1089Met) variant reduced catalytic activity in vitro, suggesting pathogenicity; c.2254C>T (p.Arg752Cys) did not reduce catalytic activity in vitro (ref. ^8^), suggesting non-pathogenicity.

^c^Variants previously tested in our in vitro assay (ref. ^8^).

^d^Unaffected daughter of Sample 5 and sister of Sample 4.

^e^Unaffected son of 25 and brother of 26.

^f^Unaffected mother of Sample 4.

^g^Unaffected sister of Sample 6 and daughter of Samples 11 and 14.

^h^Previously considered VUS because mother was not thought to be affected; upon further evaluation, she was noted to be affected (see text for details).

To validate the *TET3* DNA methylation signature, we used the 285 identified probes to attempt to classify the samples from the signature validation set (samples 7–16, Table 1). We first performed MDS and hierarchical clustering and found, as expected, that the four *TET3* family member controls (samples 7–10, Table 1) clustered with the set of 30 age- and sex-matched controls used for signature discovery (Fig. 2c and Supplementary Fig. 2a). The five samples with mono-allelic pathogenic *TET3* variants (samples 12–16, Table 1) clustered with the other mono-allelic pathogenic samples used for signature discovery (Fig. 2c and Supplementary Fig. 2a). The sample predicted to have a non-pathogenic (benign) *TET3* variant based on in vitro catalytic activity^8^ (sample 11, Table 1) clustered with other controls as expected on hierarchical clustering (Supplementary Fig. 2a) and between the mono-allelic pathogenic and control samples on MDS (Fig. 2c), suggesting that it may have a minimal effect on DNA methylation.

We next used the set of 285 probes and the signature discovery samples with their matching controls to train a support vector machine (SVM) to classify the sample types. The model was set to generate methylation variant pathogenicity (MVP) prediction scores from 0 to 1, with a higher score representing a greater chance that the sample has a methylation signature similar to the *TET3* episignature. Using this model, we generated MVP scores for the training set of signature discovery samples along with the validation samples. The *TET3* bi-allelic samples had the highest scores (>0.95), controls all had scores near zero, and the mono-allelic samples had more moderate scores from approximately 0.5–0.8 (Fig. 2d). Importantly, the validation set scores reflected their expected pathogenicity status. The predicted benign sample again had a score between controls and mono-allelic samples (Fig. 2d).

### The *TET3* episignature can be used to classify variants of uncertain significance

Having validated the initial *TET3* episignature by confirming its ability to correctly categorize the validation samples, including four family member controls and five with mono-allelic pathogenic *TET3* variants, we added the latter five samples to the original training set of six discovery samples and repeated the analysis for signature discovery to generate a more robust *TET3* episignature. Using these 11 *TET3* samples and a set of 55 unrelated age-matched and sex-matched controls (five control samples for every *TET3*-deficient sample), we identified 2960 probes with a mean methylation difference of at least 10% between the *TET3* and control samples, 23,610 probes with an adjusted *p* value < 0.001, and 1211 probes fulfilling both criteria. After ROC analysis and correlation filtering we obtained a final list of 677 probes (Supplementary Fig. 2b), 673 of which had increased DNA methylation, comprising DNA methylation signature 2. Of note, 141 of the 677 probes were also found in the first episignature (Supplementary Fig. 3).

**Fig. 3:**
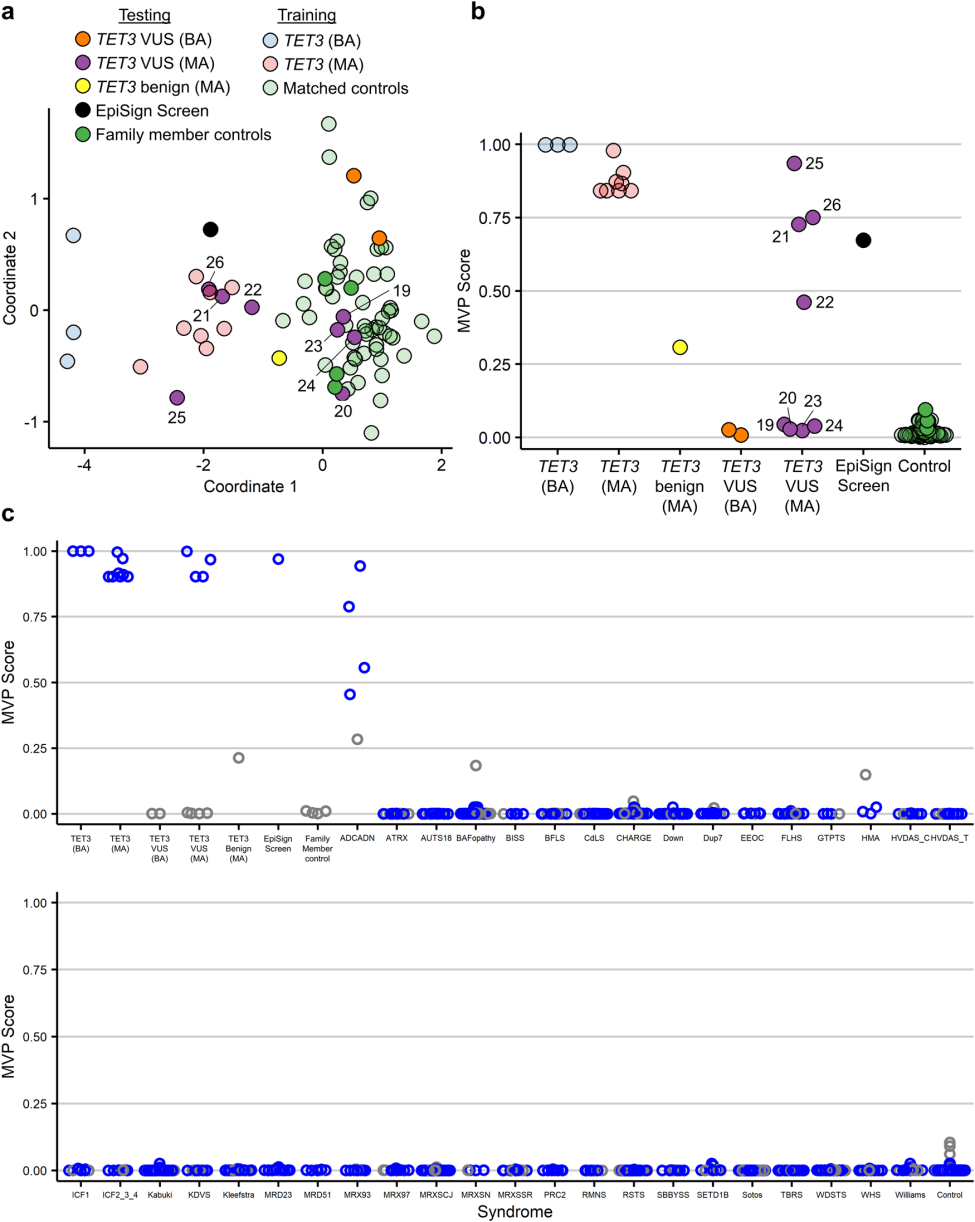
Classification of samples using the *TET3* episignature. **a** Multidimensional scaling (MDS) plot of the *TET3* signature discovery, validation, and testing samples, including one previously unknown sample identified from the EpiSign database, using the episignature from the second round of signature discovery. The discovery and validation pathogenic samples (*n* = 3 *TET3* (BA), light blue; *n* = 8 *TET3* (MA), light red) along with matched controls (*n* = 55, light green) were used to identify a *TET3* episignature, which was then used to classify the remaining samples, including the VUS’s of the testing cohort (*n* = 2 *TET3* VUS (BA), orange; *n* = 8 *TET3* VUS (MA), purple; *n* = 1 *TET3* benign (MA), yellow; *n* = 1 EpiSign screen, black; *n* = 4 family member controls, green). The 66 samples used for signature identification are shown as partially transparent circles, and the remaining samples are opaque. See Table 1 for descriptions of samples. *TET3* VUS (MA) samples are numbered according to Table 1. **b** Methylation variant pathogenicity (MVP) plot of the same samples. **c** The *TET3* episignature from the final round of signature discovery applied to samples from 46 other neurodevelopmental conditions, which exhibit 38 different DNA methylation episignatures in our EpiSign database (some syndromes share signatures). For each syndrome and for control samples, 75% of samples were used to train the classifier (blue) and 25% were used for testing (gray). VUS, variants of uncertain significance; *TET3* VUS (BA), samples with bi-allelic *TET3* VUS’s; *TET3* VUS (MA), samples with mono-allelic *TET3* VUS’s; *TET3* benign (MA), the benign variant that did not reduce catalytic activity in vitro^8^; EpiSign screen, an unknown sample identified by screening the Episign database; family member controls, family members of affected individuals lacking *TET3* variants; *TET3* (BA), samples with bi-allelic pathogenic *TET3* variants; *TET3* (MA), samples with mono-allelic pathogenic *TET3* variants; matched controls, age-matched and sex-matched controls. Syndrome abbreviations: ADCADN, Autosomal dominant cerebellar ataxia, deafness, and narcolepsy; ATRX, Alpha-thalassemia mental retardation syndrome; AUTS18, Autism, susceptibility to, 18; BAFopathy, Coffin-Siris 1–4,8 (CSS1–4,8) & Nicolaides-Baraitser (NCBRS) syndromes; BISS, Blepharophimosis Intellectual disability SMARCA2 Syndrome; BFLS, Börjeson–Forssman–Lehmann syndrome; CdLS, Cornelia de Lange syndrome; CHARGE, CHARGE syndrome; Down, Down syndrome; Dup7, Williams-Beuren region duplication syndrome (Chr7q11.23 duplication syndrome); EEOC, epileptic encephalopathy, childhood-onset; FLHS, Floating-Harbor syndrome; GTPTS, Genitopatellar syndrome; HMA, Hunter-McAlpine syndrome; HVDAS_C, Helsmoortel-van der Aa syndrome (ADNP syndrome [Central region methylation signature]); HVDAS_T, Helsmoortel-van der Aa syndrome (ADNP syndrome [Terminal regions methylation signature]); ICF1, Immunodeficiency-centromeric instability-facial anomalies syndrome Type 1; ICF2_3_4, Immunodeficiency-centromeric instability-facial anomalies syndrome Types 2, 3, 4; Kabuki, Kabuki syndrome 1 and 2; KDVS, Koolen de Vreis syndrome; Kleefstra, Kleefstra syndrome; MRD23, mental retardation autosomal dominant 23; MRD51, mental retardation autosomal dominant 51; MRX93, mental retardation X-linked 93; MRX97, mental retardation X-linked 97; MRXSCJ, mental retardation X-linked, syndromic, Claes-Jensen type; MRXSN, mental retardation X-linked syndromic Nascimento-type; MRXSSR, mental retardation X-linked Snyder-Robinson type; PRC2, PRC2 complex (Weaver syndrome and Cohen-Gibson syndrome); RMNS, Rahman syndrome; RSTS, Rubinstein-Taybi syndrome; SBBYSS, Ohdo syndrome, SBBYSS variant; SETD1B, SETD1B-related syndrome; Sotos, Sotos syndrome; TBRS, Tatton-Brown-Rahman syndrome; WDSTS, Wiedemann-Steiner syndrome; WHS, Wolf-Hirschhorn syndrome; Williams, Williams-Beuren syndrome (Chr7q11.23 deletion syndrome).

DNA methylation episignatures can be powerful tools to classify VUS’s as being likely benign or pathogenic, depending on whether the DNA methylation signature in the individual with the VUS matches a known signature^11^. We therefore applied the updated *TET3* episignature to our cohort of samples with *TET3* VUSs (testing samples, 17–26, Table 1) and classified the samples using unsupervised (MDS and hierarchical clustering) and supervised (MVP score) methods. We found that the bi-allelic variants in samples 17 and 18 are likely benign, as evidenced by their clustering with control samples and having prediction scores near zero (Fig. 3a, b and Supplementary Fig. 2b). Similar results were observed in samples from the four carrier parents of individuals 17 and 18, in whom these variants were present in mono-allelic form (samples 19, 20, 23, and 24; Fig. 3a, b, Supplementary Fig. 2b). The other four mono-allelic variants (samples 21, 22, 25, and 26) clustered with known pathogenic mono-allelic samples (Fig. 3a, b and Supplementary Fig. 2b). Three of these samples (21, 25, and 26) had prediction scores between 0.75 and 1, as we typically see for pathogenic variants, while the fourth sample (22) had a more moderate score of 0.46 (Fig. 3b). Considering all the evidence together for this variant—the MDS (Fig. 3a) and the hierarchical clustering (Supplementary Fig. 2b) along with the MVP score (Fig. 3b)—clearly points toward the variant present in sample 22 being pathogenic.

### Previously unreported individuals with pathogenic variants identified using the *TET3* episignature

Using DNA methylation profiling of whole blood, we have identified and confirmed eight additional individuals from five families with pathogenic variants in *TET3* (Supplementary Data 2) in our signature validation (Fig. 2c, d) and testing (Fig. 3a, b) cohorts. All individuals harbor mono-allelic variants and were referred to our study due to the presence of a suspected pathogenic *TET3* variant. Importantly and distinct from the other affected individuals, Individual 5-1 (Supplementary Data 2; sample 27 from Table 1) was identified exclusively via DNA methylation profile by using the *TET3* episignature to screen the EpiSign database which contains over 1000 samples from individuals without a previous genetic diagnosis (Fig. 3a–c, EpiSign Screen). After identifying a BEFAHRS episignature in individual 5-1 (sample 27), follow-up analysis of the previously generated exome data in this individual revealed a mono-allelic nonsense variant (c.738C>A; p.Cys246*) in him (Fig. 3a, b, Episign screen) and his potentially mosaic mother (Individual 5-II, Supplementary Data 2). The eight additional cases from five distinct lineages reported here share clinical features with the original eight reported families^8^ (Supplementary Data 2; and Supplementary note), specifically ID (6/8), developmental delay (6/8), autistic traits (5/8), and facial dysmorphisms (7/8) (Supplementary Fig. 4). These cases had a wide range of severity, with variable expressivity noted. Interestingly, hypotonia (2/8) and growth abnormalities (2/8) were less common than previously reported^8^. However, a proband with predominant psychiatric manifestations suggests expansion of the phenotype. Together, these additional cases help further delineate the phenotypic spectrum associated with pathogenic variants in *TET3* leading to BEFAHRS and demonstrate the utility of Episign to support genetic diagnosis, especially in diseases without highly specific manifestations.

### The *TET3* episignature differentiates BEFAHRS from other neurodevelopmental and congenital anomaly syndromes

We performed a final round of episignature discovery (Supplementary Fig. 5a) by adding the five samples with mono-allelic *TET3* VUS’s that were reclassified as likely pathogenic (samples 21, 22, 25–27, Table 1). Using this new training set of 16 *TET3* samples and 64 unrelated age-matched and sex-matched controls (four controls for every *TET3-*deficient sample), we identified 2054 probes with a mean methylation difference of at least 10% between the *TET3*-deficient and control samples, 29,813 probes with an adjusted p value <0.001, and 1094 probes fulfilling both criteria. After ROC analysis and correlation filtering we obtained a final list of 567 probes, all of which had increased DNA methylation (Supplementary Fig. 5a). 418 of the 567 probes were also found in the second signature (Supplementary Fig. 3). Despite somewhat less clear separation between bi-allelic and mono-allelic pathogenic samples and the “benign” sample associating with pathogenic samples on hierarchical clustering (Supplementary Fig. 5a), MDS continued to reveal three distinct groups—bi-allelic pathogenic, mono-allelic pathogenic, and controls—with the benign variant again clustering between the latter two (Supplementary Fig. 5b).

We have previously demonstrated the ability of using DNA methylation episignatures to differentiate between multiple neurodevelopmental and congenital anomaly syndromes^10,28^. To determine whether BEFAHRS could also be differentiated, we used a multi-class prediction model to compare the *TET3*-deficient samples with samples from 46 other neurodevelopmental conditions with 38 distinct DNA methylation episignatures in the EpiSign database^10^ and from additional controls (Fig. 3c). In addition to the TET3 and related samples (all samples in Table 1) and the 64 unrelated age-matched and sex-matched controls used for signature discovery (probe selection), this plot also includes 549 additional unrelated controls and over 1000 samples from other syndromes used to train (75% of these samples) and test (25% of these samples) the classifier. While the *TET3*-deficient samples were all analyzed using the EPIC array, which contains over 850,000 probes, many of the other samples were analyzed using Illumina’s 450K array, which contains about half as many probes. We therefore removed from the 567 *TET3*-specific probes any probes which are not also found on the 450K array, leaving 346 probes.

The classification model was retrained using *TET3*-deficient samples against samples from other syndromes along with controls (instead of only using controls) in a one-against-all approach^10^. For each syndrome, 75% of samples were used to train the classifier, and 25% were kept for testing. We found that the *TET3*-deficient samples had probability scores similar to our previous analysis (Fig. 3c). All control samples and samples from individuals with other syndromes, with one exception, had scores near 0, indicating that their methylation signatures can be successfully distinguished from the *TET3* signature (Fig. 3c). Interestingly, samples from patients diagnosed with ADCADN (MIM: 604121) had higher scores. ADCADN is caused by mutations, which are thought to be activating^29^, in *DNMT1*^30^, a DNA methyltransferase with a molecular function opposite to that of TET3.

**Fig. 4:**
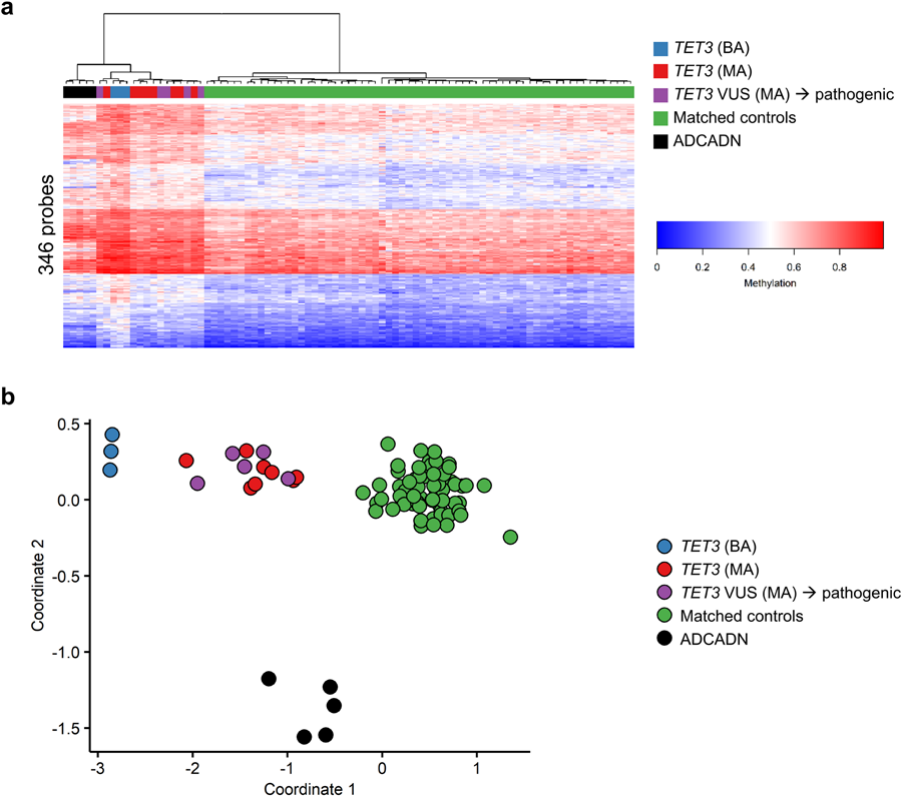
ADCADN and *TET3*-deficient samples can be distinguished based on unique DNA methylation patterns. **a** Hierarchical clustering of ADCADN (*n* = 5, black), *TET3*-deficient (*n* = 3 *TET3* (BA), blue; *n* = 8 *TET3* (MA), red; *n* = 5 *TET3* VUS (MA) → pathogenic, purple), and matched control (*n* = 64, green) samples. Each row of the heatmap represents one CpG probe, and each column represents one individual’s sample. The heatmap color scale from blue to red represents the DNA methylation level (beta value) from 0 (no methylation) to 1 (fully methylated). Because the ADCADN samples were analyzed using 450K arrays and the *TET3-*deficient and control samples were analyzed using EPIC arrays, this plot was generated using the 346 probes in the *TET3* episignature that are common between the EPIC and 450K arrays. **b** Multi-dimensional scaling (MDS) plot shows that the *TET3* episignature can differentiate between *TET3-*deficient and ADCADN samples. Color coding and numbers of samples are the same as in **a**. ADCADN, autosomal dominant cerebellar ataxia, deafness, and narcolepsy; VUS, variant of uncertain significance; *TET3* (BA), samples with bi-allelic pathogenic *TET3* variants; *TET3* (MA), samples with mono-allelic pathogenic *TET3* variants; *TET3* VUS (MA) → pathogenic, samples with mono-allelic *TET3* VUS’s re-classified as pathogenic; matched controls, age-matched and sex-matched controls.

To investigate the relationship between methylation changes in samples with *TET3* and *DNMT1* mutations, we repeated hierarchical clustering (Fig. 4a) and MDS analysis (Fig. 4b) with the same set of 16 *TET3-*deficient samples and 64 age-matched and sex-matched controls (four controls for every *TET3-*deficient sample) as used above in the final round of episignature discovery but with the addition of the *DNMT1* samples. This analysis used the 346 probes identified after the final round of *TET3* episignature training. Both of these methods of unsupervised clustering show that the ADCADN samples do not cluster with the *TET3* samples (Fig. 4a, b). However, at a subset of TET3 DMRs there is a similar trend between these disease states: the ADCADN samples exhibited increased DNA methylation compared to controls at 34 of the 50 DMRs, while TET3 samples exhibited increased methylation at all 50 (Supplementary Fig. 6). Therefore, while there may be some overlap in the methylation changes between the two sets of samples, they can still be distinguished using a combination of supervised and unsupervised classification systems. Overall, the *TET3* episignature can successfully distinguish individuals with pathogenic variants in *TET3* from individuals with 46 other neurodevelopmental and congenital anomaly syndromes, including ADCADN.

## Discussion

In this work, we have identified a robust genome-wide DNA methylation signature in whole blood, which differentiates pathogenic from non-pathogenic variants in *TET3*, has greatly aided in the rapid characterization of this newly-described neurodevelopmental disorder, and will be of critical value to facilitate ongoing diagnosis of BEFAHRS with its non-specific phenotypic features. The *TET3* episignature is unique because it provides a quantitative and functional readout of TET activity, and appears to be dose-dependent based on the amount of residual TET3 activity. It most strikingly differentiates affected individuals with proven bi-allelic hypomorphic variants from controls but also stratifies affected individuals into three distinct groups based on molecular subtype—bi-allelic, mono-allelic, and control. There are a few reports of Mendelian disorders of the epigenetic machinery with mixed autosomal dominant and autosomal recessive inheritance patterns^31,32^, but we are not aware of any with established DNA methylation signatures for comparison. Our observed “dosage effect” is reminiscent of those observed in individuals with deletions and duplications of 7q11.23^33^ and with Claes-Jensen syndrome (MIM: 300534), an X-linked form of ID, in which severely affected male individuals have a distinct DNA methylation signature that distinguishes them from unaffected controls, and both groups can be differentiated from female unaffected or mildly affected carriers with an intermediate signature^12^.

Another illustration of the quantitative, dose-dependent nature of the identified *TET3* signature comes from variants identified in a single family reported previously (Family 1)^8^ in which the proband had bi-allelic variants inherited in trans from her parents with the maternal variant reducing catalytic activity and the paternal variant not reducing catalytic activity in our in vitro assay^8^. We therefore categorized the variants as pathogenic and non-pathogenic, respectively, and re-classified the proband as an individual with a mono-allelic pathogenic variant (sample 6, Table 2). Not surprisingly, her DNA methylation profile—and milder phenotypic features—more closely resembled that of other individuals with mono-allelic variants, supporting her classification as “functionally mono-allelic”. Also supporting this, her mother, who shares the hypomorphic c.3265G>A (p.Val1089Met) variant, has phenotypic features of anxiety, depression, and possible attention deficit hyperactivity disorder (ADHD), and had a DNA methylation profile similar to her daughter and to other individuals with mono-allelic pathogenic variants (sample 14, Table 2). Based on the mother’s mild presentation and our analysis, autosomal dominant inheritance with variable expressivity should be considered. However, we cannot rule out the possibility that the paternally-inherited variant (c.2254C>T; p.Arg752Cys)—which did not reduce catalytic activity in vitro^8^ but which produces an episignature intermediate between controls and mono-allelic affected individuals in most analyses—is contributing to the proband’s phenotype (sample 11, Table 2).

**Table 2:** Variant pathogenicity prediction.

Sample	Used for	Sample type/ Predicted pathogenicity	TET3 amino acid changes	CADD score	GnomAD alleles	Inheritance	In catalytic domain?	Evidence supporting pathogenicity	Evidence against pathogenicity	Epi-signature pathogenicity prediction
1^a^	Signature discovery	*TET3* (BA); path	p.Phe1072Cys^c^; p.Ala1076Thr^c^	28.2; 25.9	0; 0	AR, Cpd het	Yes; Yes	Both low TET activity^i^; CADD; gnomAD; inheritance; in cat domain	None	Both path
2^a^	Signature discovery	*TET3* (BA); path	p.Val908Leu; p.Val908Leu^c^	27	0	AR, Hom	Yes	Low TET activity^i^; CADD; gnomAD; inheritance; in cat domain	None	Path
3^a^	Signature discovery	*TET3* (BA); path	p.Val908Leu; p.Val908Leu^c^	27	0	AR, Hom	Yes; Yes	Low TET activity^i^; CADD; gnomAD; inheritance; in cat domain	None	Path
4^a^	Signature discovery	*TET3* (MA); path	p.His1660Profs*52	NA	0	AD, inherited	Yes	Predicted LOF; gnomAD; inheritance; in cat domain	None	Path
5^a^	Signature discovery	*TET3* (MA); path	p.His1660Profs*52	NA	0	Unkn	Yes	Predicted LOF; gnomAD; in cat domain	None	Path
6^a^	Signature discovery	*TET3* (MA^b^); path	p.Val1089Met^c^; p.Arg752Cys^c^	29.1; 23.6	0; 29 (0 hom)	Initially AR; now AD^b^	Yes; No	p.Val1089Met low TET activity^i^; CADD; gnomAD; in cat domain. p.Arg752Cys CADD	p.Val1089Met none; p.Arg752Cys nl TET activity^i^; gnomAD; not in cat domain	Path/Intermed
7	Signature validation	Family ctl^d^; NA	Family variant absent (Sanger seq)	NA	NA	NA	NA	NA	NA	Non-path
8	Signature validation	Family ctl^e^; NA	Family variant absent (Sanger seq)	NA	NA	NA	NA	NA	NA	Non-path
9	Signature validation	Family ctl^f^; NA	Family variant absent (Exome seq)	NA	NA	NA	NA	NA	NA	Non-path
10	Signature validation	Family ctl^g^; NA	Family variants absent (Sanger, exome seq)	NA	NA	NA	NA	NA	NA	Non-path
11	Signature validation	*TET3* (MA); benign	p.Arg752Cys^c^	23.6	29 (0 hom)	Unkn	No	CADD	Nl TET activity^i^; gnom-AD; not in cat domain	Intermed
12^a^	Signature validation	*TET3* (MA); path	p.Gln1695*	44	0	AD, de novo	Yes	Predicted LOF; CADD; gnomAD; inheritance; in cat domain	None	Path
13^a^	Signature validation	*TET3* (MA); path	p.Arg1034*	38	0	AD, de novo	Yes	Predicted LOF; CADD; gnomAD; inheritance; in cat domain	None	Path
14^a^	Signature validation	*TET3* (MA); path	p.Val1089Met^c^	29.1	0	Unkn	Yes	Low TET activity^i^; CADD; gnomAD; in cat domain.	None	Path
15^a^	Signature validation	*TET3* (MA); path	p.Val908Leu^c^	27	0	Unkn	Yes	Low TET activity^i^; CADD; gnomAD; in cat domain	None	Path
16^a^	Signature validation	*TET3* (MA); path	p.Ala1076Thr^c^	25.9	0	Unkn	Yes	Low TET activity^i^; CADD; gnomAD; in cat domain	None	Path
17	Testing	*TET3* (BA) VUS; unkn	p.Pro495Ser; p.Val1295Ile	7.9; 19	13 (0 hom); 127 (0hom)	AR, Cpd het	No; Yes	p.Pro495Ser none p.Val1295Ile in cat domain	Both CADD; gnomAD; p.Pro495Ser not in cat domain	Both likely benign
18	Testing	*TET3* (BA) VUS; unkn	p.Gly1505Arg; p.Trp1746Ser	24.5; 29.3	3 (0 hom); 61 (0 hom)	AR, Cpd het	Yes; Yes	CADDs; both in cat domain	GnomAD; both present in unaffected sib	Both likely benign
19	Testing	*TET3* (MA) VUS; unkn	p.Pro495Ser	7.9	13 (0 hom)	Unkn	No	None	CADD; gnomAD; not in cat domain	Likely benign
20	Testing	*TET3* (MA) VUS; unkn	p.Val1295Ile	19	127 (0 hom)	Unkn	Yes	In cat domain	CADD; gnomAD	Likely benign
21^a^	Testing	*TET3* (MA) VUS; unkn	p.Arg911Gln	27.1	0	AD, de novo	Yes	CADD; gnomAD; inheritance; in cat domain	None	Likely path
22^a^	Testing	*TET3* (MA) VUS; unkn	p.Arg1683His	31	0	AD, de novo	Yes	CADD; gnomAD; inheritance; in cat domain	None	Likely path
23	Testing	*TET3* (MA) VUS; unkn	p.Gly1505Arg	24.5	3 (0 hom)	Unkn	Yes	CADD; in cat domain	GnomAD; this variant, p.Trp1746Ser in unaffected sib	Likely benign
24	Testing	*TET3* (MA) VUS; unkn	p.Trp1746Ser	29.3	61 (0 hom)	Unkn	Yes	CADD; in cat domain	GnomAD; this variant, p.Gly1505Arg in unaffected sib	Likely benign
25^a^	Testing	*TET3* (MA) VUS; unkn	p.Thr680Tyrfs*26^h^	NA	0	Unkn	No	Predicted LOF; gnomAD; inheritance-segregates with phenotype in family	None	Likely path
26^a^	Testing	*TET3* (MA) VUS; unkn	p.Thr680Tyrfs*26^h^	NA	0	AD, inherited	No	Predicted LOF; gnomAD; inheritance-segregates with phenotype in family	None	Likely path
27^a^	Testing	Episign screen; unkn	p.Cys246*	34	0	AD, inherited	No	Predicted LOF; CADD; gnomAD; inherited from mosaic mother	None	Likely path

*BA* bi-allelic, *MA* mono-allelic, *path* pathogenic, *non-path* non-pathogenic, *NA* not applicable, *unkn* unknown, *AR* autosomal recessive, *Cpd het* compound heterozygote, *Hom* homozygous, *AD* autosomal dominant, *LOF* loss-of-function, *cat* catalytic, *nl* normal, *seq* sequencing, *sib* sibling, *CADD score* combined annotation-dependent depletion score (https://cadd.gs.washington.edu/); gnomAD browser is at https://gnomad.broadinstitute.org/.

^a^TET3-deficient pathogenic samples used to identify the final DNA methylation episignature.

^b^Considered mono-allelic because only the c.3265G>A (p.Val1089Met) variant reduced catalytic activity in vitro, suggesting pathogenicity; c.2254C>T (p.Arg752Cys) did not reduce catalytic activity in vitro, suggesting non-pathogenicity (ref. ^8^).

^c^Variants previously tested in our in vitro assay (ref. ^8^).

^d^Unaffected daughter of Sample 5 and sister of Sample 4.

^e^Unaffected son of 25 and brother of 26.

^f^Unaffected mother of Sample 4.

^g^Unaffected sister of Sample 6 and daughter of Samples 11 and 14.

^h^Previously considered VUS because mother was not thought to be affected; upon further evaluation, she was noted to be affected (see text for details).

^i^TET activity based on in vitro assay performed and reported in reference^8^.

In addition, the *TET3* episignature was able to accurately reclassify VUS’s based on their DNA methylation profiles (Table 2). The c.2036dupC variant that results in a frameshift (p.Thr680Tyrfs*26; samples 25 and 26, Table 2) was initially characterized as a VUS because the mother from whom the variant was inherited was reported to be unaffected. However, the methylation profile and the frameshift nature of the variant strongly supported pathogenicity. Reclassification of the variant as pathogenic was ultimately confirmed upon receipt of new phenotypic information that the mother in fact had ID, anxiety, and depression, and attended a special needs school, as well as segregation studies using Sanger sequencing showing that the variant tracked with ID and other features of BEFAHRS in the three affected individuals in the family (Supplementary Data 2; Family 1). Similarly, for individual 18 (Table 2) with bi-allelic variants inherited in trans from unaffected carrier parents (c.4513G>A; p.Gly1505Arg and c.5237G>C; p.Trp1746Ser), both were initially considered VUS’s. Here, we identified a DNA methylation profile similar to control individuals in the proband and both parents. Simultaneously, during the course of our studies, segregation analysis by Sanger sequencing revealed that an unaffected sister shared both variants with the severely affected proband, making it highly unlikely that these *TET3* variants are disease-causing and supporting the observed DNA methylation profile, which is similar to controls (Table 2). Furthermore, this example illustrates that not all variants in *TET3* (but rather just pathogenic ones) lead to the BEFAHRS methylation signature identified here.

In addition to the above examples in which segregation studies supported the predictions of DNA methylation profiles in the determination of variant pathogenicity, other metrics also helped to validate the use of episignatures in variant classification. Combined annotation-dependent depletion (CADD) scores, the presence of variants in gnomAD (signifying their presence in healthy controls), inheritance patterns, and the protein domain location of each missense variant were analyzed (Table 2). For all VUS’s analyzed as part of the testing cohort—and for all variants—the combined evidence for or against pathogenicity always supported the DNA methylation profile prediction, suggesting that the episignature was in fact able to correctly characterize each variant as pathogenic/likely pathogenic or likely benign (Table 2). This was even true for the Arg752Cys variant—which has an intermediate DNA methylation signature between mono-allelic pathogenic and control samples but more often resembles controls. Putting all variant classifying information together, this variant would be classified as likely benign.

Remarkably, the *TET3* DNA methylation profile was able to go beyond successful classification of *TET3* variants, including VUS’s, to identify a case of BEFAHRS that was not previously suspected. Individual 27 (Table 2; 5-I and Supplementary Data 2) had previously had negative trio exome sequencing and was undergoing further genetic evaluation using the clinically available Episign test for suspected CHARGE syndrome or Kabuki syndrome based on the findings of congenital heart disease (Tetralogy of Fallot), small size, borderline developmental delay, and craniofacial features. The Episign test ultimately came back negative for the suspected disorders, and the 44 other conditions that are currently part of clinical EpiSign testing^10^ (https://genomediagnostics.amsterdamumc.nl/product/episign-complete/). However, reanalysis using the newly discovered *TET3* episignature identified this sample as positive (or *TET3*-deficient). Upon subsequent reanalysis of the previous exome data generated prior to the initial reporting of BEFAHRS/*TET3* deficiency^8^, a nonsense variant in *TET3* was in fact identified in the proband and in his more mildly affected—and potentially mosaic—mother, who had facial features consistent with BEFAHRS and social difficulties in childhood. Using the *TET3* DNA methylation signature to diagnose an individual not previously suspected of having BEFAHRS emphasizes the robustness and specificity of the signature. It also illustrates the utility of DNA methylation arrays in making a diagnosis in previously undiagnosed individuals with non-specific features and supports the use of DNA methylation arrays early on in the diagnostic work up for developmental disorders and multiple congenital anomaly syndromes, particularly when a Mendelian disorder of the epigenetic machinery is suspected^10,20,34^.

The *TET3* episignature was ultimately able to differentiate individuals with pathogenic *TET3* variants from individuals with 46 other syndromes having 38 distinct DNA methylation episignatures. However, as we have previously shown, this becomes more challenging with increasing numbers of conditions and when those conditions (and their corresponding methylation patterns) overlap^10,11^. Using the *TET3* episignature generated by training the *TET3* cohort samples against controls and 38 other episignatures and our supervised classification algorithm, we observed high MVP scores partially overlapping those of *TET3*-deficient samples for individuals with ADCADN, which results from mutations in the DNA methyltransferase writer *DNMT1*^30^. This indicates that, at least for the set of probes used to generate the episignature, these ADCADN samples exhibit a trend toward DNA hypermethylation, similar to *TET3-*deficient samples. This fits with our previous findings showing that when hierarchical clustering is used to compare all the syndromes for which we have episignatures, syndromes tend to cluster based on their overall global hypomethylation or hypermethylation status^10^. However, similar to our previous results in other syndromes^10,11^, when ADCADN samples and controls were included in the hierarchical clustering and MDS analysis along with *TET3-*deficient samples and controls, we were able to clearly differentiate the two disorders based on their unique DNA methylation profiles. Whereas most of the episignature overlap between the two disorders is likely accounted for by generalized hypermethylation of DNA, we observed trends toward similar differential (increased) methylation patterns at specific regions (Supplementary Fig. 6).

The observation that BEFAHRS and ADCADN have highly similar DNA methylation profiles favoring hypermethylation may be reflective of the biological function of the corresponding proteins. The episignature for ADCADN was generated using samples from a family with the same recurrent mutation, c.1709C>T in exon 21, which leads to the p.Ala570Val missense variant in the replication foci targeting sequence (RFTS) domain^13^, and similar nearby missense variants in other individuals with ADCADN have been shown to prevent inhibition of DNMT1 activity, thereby increasing DNA methylation^29^. This fits with our observation here and previously^13^ of a trend toward an overall increase in DNA methylation in whole blood from individuals with ADCADN. Similarly, here we observed a genome-wide increase in DNA methylation in individuals with hypomorphic variants in the *TET3* eraser of 5mC. The observations that gain of DNMT1 writer activity and loss of TET3 eraser activity both lead to hypermethylation of DNA make sense and are consistent with our previously proposed Balance hypothesis suggesting that opposing writers and erasers of particular epigenetic marks are present at target genes (or other genomic regions), and that any disruption would lead to changes in levels of relevant epigenetic marks and have additional downstream consequences on chromatin structure and gene expression^1^.

Here, we report eight previously undescribed individuals from five families with BEFAHRS, increasing the number of individuals described in the literature and confirming the phenotype. Similar to our previous report, all individuals, with the exception of one family reported here, exhibit global developmental delay and/or ID. The above findings confirm that BEFAHRS, like other Mendelian disorders of the epigenetic machinery, is highly associated with additional neurobehavioral features, including autism and difficulties with social interactions, seizures and EEG abnormalities, anxiety, ADHD, and in some cases depression. The affected individuals reported here have similar facial features to those reported previously, including tall and broad foreheads and long, hypotonic faces^8^. Based on the current and previous^8^ reports, we propose the following mnemonic for BEFAHRS (MIM: 618798): **B**ehavioral differences, **E**pilepsy, characteristic **F**acial features, **A**utistic features, **H**ypotonia, **R**etardation of psychomotor development, and **S**ize differences.

This report not only confirms but also expands the BEFAHRS phenotype. Here, we report one male proband who presented with acute psychiatric symptoms associated with cognitive decline as an adolescent. His features included depression, severe anxiety with panic attacks, and periods of psychosis with hallucinations, aggression, and self-mutilation alternating with periods of normal behavior; he may have had mild features of developmental delay as a child as well, which were only recognized later. Whereas this type of presentation has not been described previously in an affected proband with BEFAHRS, these findings are remarkably similar to those observed in a previously reported carrier mother from a consanguineous family^8,9^. She exhibited severe anxiety, psychosis, and difficulties with short-term memory but was not brought to medical attention until her three adult children with ID due to a hypomorphic homozygous missense variant in *TET3* were identified^8^. Interestingly, both of these individuals with similar and predominantly psychiatric presentations—the severely affected carrier mother^8^ and the young adult male reported here—have missense variants nearly adjacent to one another, p.Val908Leu and p.Arg911Gln, respectively. These variants constitute two of the three reported to occur within the cysteine-rich region of the catalytic dioxygenase domain of TET3, which is essential for catalytic activity. While confirmation in additional affected individuals is necessary, this observation suggests the potential for an emerging genotype–phenotype correlation between mono-allelic missense variants in this particular region and predominant psychiatric disease presentations.

Notably, analysis of individual DMRs may provide additional insight into disease pathogenesis. While it is unclear whether DMRs in whole blood reflect methylation changes in the brain, the most disease-relevant tissue, the observation that expression of the identified DMR-associated genes is significantly higher than other protein-coding genes in fetal cerebral excitatory and inhibitory neurons is intriguing, particularly because these DMRs are abnormally hypermethylated in blood of *TET3*-deficient individuals. If this hypermethylation is also present in the cerebral excitatory and inhibitory neurons of the developing fetus, it could result in abnormal silencing of these genes, potentially contributing to the pathogenesis of BEFAHRS. Further supporting biological relevance, 20 of the DMR-associated genes encoded proteins whose function, if disrupted, would be predicted to lead to one or more phenotypic features of BEFAHRS. Further analysis of these DMRs and others is planned; direct measurement and comparison of DNA methylation within regions of interest in blood and in phenotypically-relevant cells from brain will further our understanding of the role of DNA demethylation in health and disease.

In summary, here we establish a specific and robust genome-wide DNA methylation profile that has helped to refine our understanding of a novel disorder of DNA demethylation—BEFAHRS—at the molecular and phenotypic levels. As a highly sensitive and specific biomarker, the *TET3* episignature can categorize genetic variants as pathogenic or benign and diagnose individuals not previously suspected as having the disorder. In particular, the *TET3*-specific episignature can help resolve cases with ambiguous or incompletely penetrant phenotypes in this Mendelian disorder with a complex inheritance pattern. In addition, we have identified unexpected links between two disorders with partially-overlapping DNA methylation profiles and DMRs potentially relevant to disease pathogenesis. Genome-wide DNA methylation analysis has become a critical diagnostic tool with the potential to reveal mechanistic insights into the role of DNA methylation in biology. Moreover, establishment of episignatures like this one in additional Mendelian disorders of the epigenetic machinery will allow us to elucidate common disease mechanisms in and develop targeted therapies for many disorders within this rapidly expanding group.

## Methods

### Statement on ethics approval

Written informed consent was obtained from all individuals or family member legal representatives prior to inclusion in the study. Specifically, written informed consent for genome-wide analysis with exome sequencing was obtained for all individuals. Written informed consent for DNA methylation array analysis was obtained, either on a Johns Hopkins Institutional Review Board (IRB)-approved consent form and/or de-identified samples were submitted for analysis after consent was obtained locally. The study protocol has also been approved by the Western University Research Ethics Board (REB 106302). The authors affirm that human research participants provided informed consent for publication of the images in Supplementary Fig. 4. In some cases, copies of the signed consent forms were submitted with the manuscript. Alternatively, signed consent forms remain on-file at the institution of the contributing authors, and a letter from the IRB/REB confirming that consent was obtained was submitted with the manuscript in place of the original consent forms. All consent forms have been approved by the local institutional review boards (or equivalent) at the institutions of the contributing authors. Data from the Deciphering Developmental Disorders (DDD; https://www.ddduk.org/) and the MAGIC projects, approved by the Central Manchester (02/CM/238) and Cambridge South NHS REC (10/H0305/83), respectively, were used in this study.

### Patient cohort samples

The cohort consisted of individuals with bi-allelic or mono-allelic *TET3* variants predicted to be benign, pathogenic, or VUS’s; control individuals without *TET3* variants; and one individual whose *TET3* status was not previously known (Table 1). Specifically, frameshift and nonsense variants were categorized as pathogenic if de novo in the affected proband or if inherited from a parent with a similar phenotype, and missense variants were considered pathogenic if they were previously shown to reduce catalytic activity in our in vitro assay, which was the case for all missense variants labeled “pathogenic” in the discovery and validation cohorts^8^. All other variants were initially deemed VUS’s, and were later reclassified based on the identified episignature and supporting information. The “Signature discovery” cohort consisted of six individuals with bi-allelic or mono-allelic pathogenic variants in *TET3* and previously reported to have a diagnosis of BEFAHRS (*TET3* deficiency)^8^ **(**Table 1**)**. The “Signature validation” cohort consisted of samples from control individuals (unaffected family members of individuals with BEFAHRS without variants in *TET3*), an individual with a presumed non-pathogenic *TET3* variant based on inability to reduce catalytic activity in vitro^8^ (labeled “benign”), and individuals with mono-allelic presumed pathogenic *TET3* variants, including affected probands and comparatively more mildly affected carrier parents. The “Testing” cohort consisted of samples from individuals initially categorized as having VUS’s in *TET3*, including two probands with bi-allelic missense variants and their four unaffected carrier parents, two individuals with mono-allelic de novo missense variants, and two individuals with the same mono-allelic frameshift variant (a mother–son duo initially described as having disparate phenotypes). In addition, the “Testing” cohort included an individual (labeled “Episign screen”), who was initially identified using the *TET3* episignature to screen a database of over 1000 undiagnosed individuals and later found to have a nonsense variant in *TET3* on prior negative exome sequencing. All variants were verified using Mutalyzer (https://mutalyzer.nl). Each cohort included a set of unrelated age- and sex-matched controls (4–5 controls for every *TET3*-deficient sample) as described. These controls were not matched for ethnicity but are mostly Western European, as were the majority of *TET3*-deficient individuals on whom DNA methylation arrays were performed.

### Sample processing

Peripheral blood DNA was extracted using standard techniques. Bisulfite conversion was performed using the Zymo EZ-96 DNA Methylation Kit (D5004), and 500 ng of bisulfite-converted DNA was used as input to the Illumina Infinium MethylationEPIC (v1-0) BeadChip array (EPIC array). Array data were generated according to the manufacturer’s protocol. 865,918 probes were interrogated and laboratory quality control was performed using the minfi package^35^. Code for minfi can be found at http://bioconductor.org/packages/release/bioc/html/minfi.html.

### Methylation data analysis

Data analysis was performed essentially as previously described^10^. IDAT files containing methylated and unmethylated signal intensity were imported into R 3.6.2 for analysis. Normalization was performed using the Illumina normalization method with background correction using the minfi package version 1.32.0^35^. Probes with detection *p*-value >0.01, located on the X and Y chromosomes, which contained SNPs at the CpG interrogation or single nucleotide extension sites, or which are known to cross-react with other genomic locations were removed, leaving 777,162 probes, which were used for subsequent analysis. To compare the overall methylation distributions between bi-allelic and mono-allelic variants, the mean methylation difference for each probe was calculated for bi-allelic samples and controls, and for mono-allelic samples and controls. A Kolmogorov–Smirnov test was used to compare the two distributions. For distribution analysis, probes that were missing data for one or more samples were excluded, leaving 776,533 complete cases. DNA methylation signature detection was performed three times as described in the results. For each round of detection the training sample set consisted of *TET3* pathogenic samples and a set of age-matched and sex-matched controls selected from our database of previously analyzed samples^10^ using the MatchIt R package version 3.0.2^36^. Each time, principal component analysis (PCA) was performed to ensure none of the selected controls were outliers. Methylation levels (beta values) were logit transformed to *M*-values and the transformed values used for linear regression modeling using the limma package version 3.42.2^37^. Estimated blood cell proportions^38^ were added to the model matrix as confounding variables. The generated *p*-values were moderated using the eBayes function. Probes, which had a mean methylation difference of at least 10% between the *TET3* and control samples and an adjusted *p* value <0.001, were selected. The list of significant probes was further filtered using receiver’s operating curve (ROC) analysis and selecting probes with an area under the curve greater than 0.9. Lastly, we calculated the Pearson’s correlation coefficients for all probes, separately within the *TET3* and control samples, and removed probes with correlations greater than 0.8. Hierarchical clustering was performed using the heatmap.2 function using Ward’s method from the gplots R package version 3.0.4. Multidimensional scaling (MDS) was performed by scaling of the pair-wise Euclidean distances between samples. The e1071 R package version 1.7-3 was used to train a support vector machine (SVM) and for construction of a multi-class prediction model as previously described^10^. To identify DMRs we used the DMRcate package version 2.0.7^39^. We selected regions which contained a minimum of five significantly different CpGs within 1 kb, with a mean methylation difference across the region of at least 10%, and with a Fisher’s multiple comparison p value for the DMR < 0.01. DMRs were annotated using the UCSC Genome Browser Data Integrator with GENCODE V3lift37 comprehensive annotations and further characterized using UCSC Genome Browser tools (https://genome.ucsc.edu).

### Comparison of DMR-associated gene expression to the expression of other genes in neurons

We downloaded the “Expression Matrix by Cell Type” from the freely available website https://descartes.brotmanbaty.org/bbi/human-gene-expression-during-development/. This matrix contains the expression (transcripts per million; TPM) of 63,561 genes (protein-coding and non-protein-coding), in 172 cell types identified using single-cell RNA-seq during fetal development^27^. Raw data for this resource are available at dbGaP (accession number phs002003.v1.p1), and processed data are available at Gene Expression Omnibus (GEO; GSE156793)^27^. We obtained the expression of each of the genes associated with our 50 DMRs in cerebral excitatory neurons and cerebral inhibitory neurons. In cases where a DMR overlapped more than one gene, we retained the gene with the highest expression. For each of the two cell types, we then compared the expression of DMR-associated genes to the expression of all other autosomal protein-coding genes (as all DMR-associated genes were on autosomes). To obtain the ENSEMBL gene identifiers of all other autosomal protein coding genes, we used the “tx_biotype” filter from the EnsDb.Hsapiens.v75 R package; this yielded 19,236 non-DMR-associated protein-coding genes. We performed the comparison using the Wilcoxon rank-sum test (one-tailed), as implemented in the wilcox.test() function in R and found the DMR-associated genes to have higher expression (*P* = 0.01 and 0.03 for excitatory and inhibitory neurons, respectively). Finally, we repeated the same analysis using the “Proportion Matrix by Cell Type”, which contains the proportion of cells in a given cell type with greater than zero unique molecular identifier (UMI) counts for a given gene. We obtained the same result, with the DMR-associated genes having higher expression (*P* = 0.01 and 0.02 for excitatory and inhibitory neurons, respectively).

### Reporting summary

Further information on research design is available in the Nature Research Reporting Summary linked to this article.

## Supplementary information

Supplementary Information

Supplementary Data 1

Supplementary Data 2

Reporting Summary

## Data Availability

The summarized, anonymized data for each subject is described in the study. The data sets generated and/or analyzed during the current study are not publicly available due to institutional and ethics restrictions. Deidentified data can be available from the corresponding authors on reasonable request. Software used in this study is publicly available and detailed analytical methodology is as previously reported^10^. The analysis of the expression of DMR-associated genes in fetal neurons utilized the freely available resource https://descartes.brotmanbaty.org/bbi/human-gene-expression-during-development/; raw data for this resource are available at dbGaP (accession number phs002003.v1.p1), and processed data are available at Gene Expression Omnibus (GEO; GSE156793)^27^.

## Code availability

All code related to the fundamental analysis of the data is from publicly available R packages as described in the text. Packages and version numbers are: R 3.6.2, minfi 1.32.0, MatchIt 3.0.2, limma 3.42.2, gplots_3.0.4, e1071 1.7-3, DMRcate 2.0.7. Additional details are included in the “Methods” section. Code for minfi can be found at http://bioconductor.org/packages/release/bioc/html/minfi.html.The analysis of the expression of DMR-associated genes in fetal neurons was performed in R version 3.6.1. The full code is available at https://github.com/hansenlab/mdem_overlap/blob/main/beck_fahrner_analysis.R.
