# Author Correction: Deficiency of *TET3* leads to a genome-wide DNA hypermethylation episignature in human whole blood

**DOI:** 10.1038/s41525-021-00269-7

**Published:** 2021-11-24

**Authors:** Michael A. Levy, David B. Beck, Kay Metcalfe, Sofia Douzgou, Sivagamy Sithambaram, Trudie Cottrell, Muhammad Ansar, Jennifer Kerkhof, Cyril Mignot, Marie-Christine Nougues, Boris Keren, Hannah W. Moore, Renske Oegema, Jacques C. Giltay, Marleen Simon, Richard H. van Jaarsveld, Jessica Bos, Mieke van Haelst, M. Mahdi Motazacker, Elles M. J. Boon, Gijs W. E. Santen, Claudia A. L. Ruivenkamp, Marielle Alders, Teresa Romeo Luperchio, Leandros Boukas, Keri Ramsey, Vinodh Narayanan, G. Bradley Schaefer, Roberto Bonasio, Kimberly F. Doheny, Roger E. Stevenson, Siddharth Banka, Bekim Sadikovic, Jill A. Fahrner

**Affiliations:** 1 Molecular Genetics Laboratory, Molecular Diagnostics Division, London Health Sciences Centre, London, ON N6A5W9 Canada; 2 National Human Genome Research Institute, Bethesda, MD 20892 USA; 3 Division of Evolution, Infection and Genomics, School of Biological Sciences, Faculty of Biology, Medicine and Health, University of Manchester, Manchester, M13 9PL UK; 4 Manchester Centre for Genomic Medicine, St Mary’s Hospital, Health Innovation Manchester, Manchester University NHS Foundation Trust, Manchester, M13 9WL UK; 5 Department of Biochemistry, Faculty of Biological Sciences, Quaid-I-Azam University, 45320 Islamabad, Pakistan; 6 Assistance Publique-Hopitaux de Paris, Sorbonne Université, Departement de Génétique, Groupe Hospitalier Pitie-Salpetriere et Hopital Trousseau, Paris, 75651 France; 7 Department of Neuropediatrics, Armand Trousseau Hospital, Assistance Publique-Hopitaux de Paris, Paris, 75012 France; 8 Laboratoire de génétique, Hôpital Pïtié-Salpêtrière, Assistance Publique-Hopitaux de Paris, Paris, 75013 France; 9 Greenwood Genetic Center, Greenwood, SC 29646 USA; 10 Department of Genetics, University Medical Center Utrecht, Utrecht University, Utrecht, The Netherlands; 11 Section Clinical Genetics, Department Human Genetics, Amsterdam University Medical Centers, Amsterdam, The Netherlands; 12 Department of Human Genetics, Laboratory of Genome Diagnostics, Amsterdam UMC, University of Amsterdam, Meibergdreef 9, Amsterdam, Netherlands; 13 Department of Human Genetics, VU University Medical Center Amsterdam, Amsterdam UMC, van der Boechorststraat 7, 1081 BT Amsterdam, The Netherlands; 14 Department of Clinical Genetics, Leiden University Medical Center, Leiden, The Netherlands; 15 Department of Human Genetics, Amsterdam Reproduction & Development Research Institute, Amsterdam UMC, University of Amsterdam, Meibergdreef 9, Amsterdam, The Netherlands; 16 Department of Genetic Medicine, Johns Hopkins University School of Medicine, Baltimore, MD 21205 USA; 17 Department of Biostatistics, Johns Hopkins University, Baltimore, MD 21205 USA; 18 Center for Rare Childhood Disorders, Translational Genomics Research Institute, Phoenix, AZ USA; 19 University of Arkansas for Medical Sciences, Springdale, AR 72762 USA; 20 Department of Cell and Developmental Biology, University of Pennsylvania Perelman School of Medicine, Philadelphia, PA 19104 USA; 21 Epigenetics Institute, University of Pennsylvania Perelman School of Medicine, Philadelphia, PA 19104 USA; 22 Center for Inherited Disease Research, Johns Hopkins University School of Medicine, Baltimore, MD USA; 23 Department of Pathology and Laboratory Medicine, Western University, London, ON N6A5W9 Canada; 24 Department of Pediatrics, Johns Hopkins University School of Medicine, Baltimore, MD 21205 USA

**Keywords:** Epigenomics, DNA methylation, Neurodevelopmental disorders, Diagnostic markers, Neurodevelopmental disorders

Correction to: *npj Genomic Medicine* 10.1038/s41525-021-00256-y, published online 8 November 2021

In this article the author name Siddharth Banka was incorrectly written as Sidharth Banka. The original article has been corrected.

