# Supplementary material for: Deficiency of TET3 leads to a genome-wide DNA hypermethylation episignature in human whole blood
Source: NPJ Genom Med. 2021 Nov 8;6:92. doi: 10.1038/s41525-021-00256-y (PMC8576018; doi:10.1038/s41525-021-00256-y)
Supplement: Supplementary file 1 — Supplementary Information [file 41525_2021_256_MOESM1_ESM.pdf]

Supplementary Figure 1

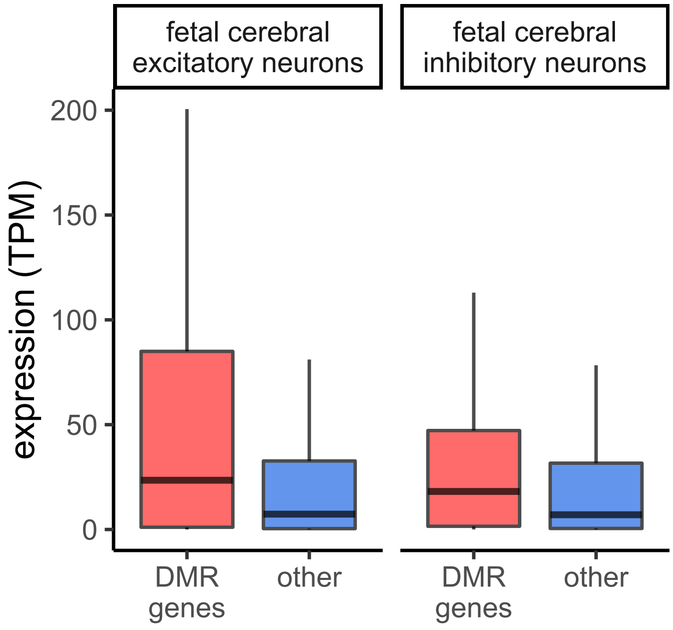

Supplementary Figure 1. Comparison of DMR-associated gene expression to other protein-coding genes in human fetal neurons. Using single-cell RNA-Seq data from human fetal cerebral excitatory and inhibitory neurons[1], expression of genes associated with the 50 most differentially-methylated DMRs was compared to expression of all other autosomal protein-coding genes and was found to be significantly higher in DMR-associated genes (see Methods for details). This was more pronounced in excitatory (median expression = 23.5 vs 7.3) compared to inhibitory neurons (median expression = 18.1 vs 7.0). For the Box-plot, center line, median; box limits, lower quartile (Q1) and upper quartile (Q3); whiskers, 1.5x interquartile range. One-tailed Wilcoxon rank-sum test, $P = 0.01$ and 0.03 for excitatory and inhibitory neurons, respectively; TPM, transcripts per million; DMR, differentially-methylated region.

Supplementary Figure 2

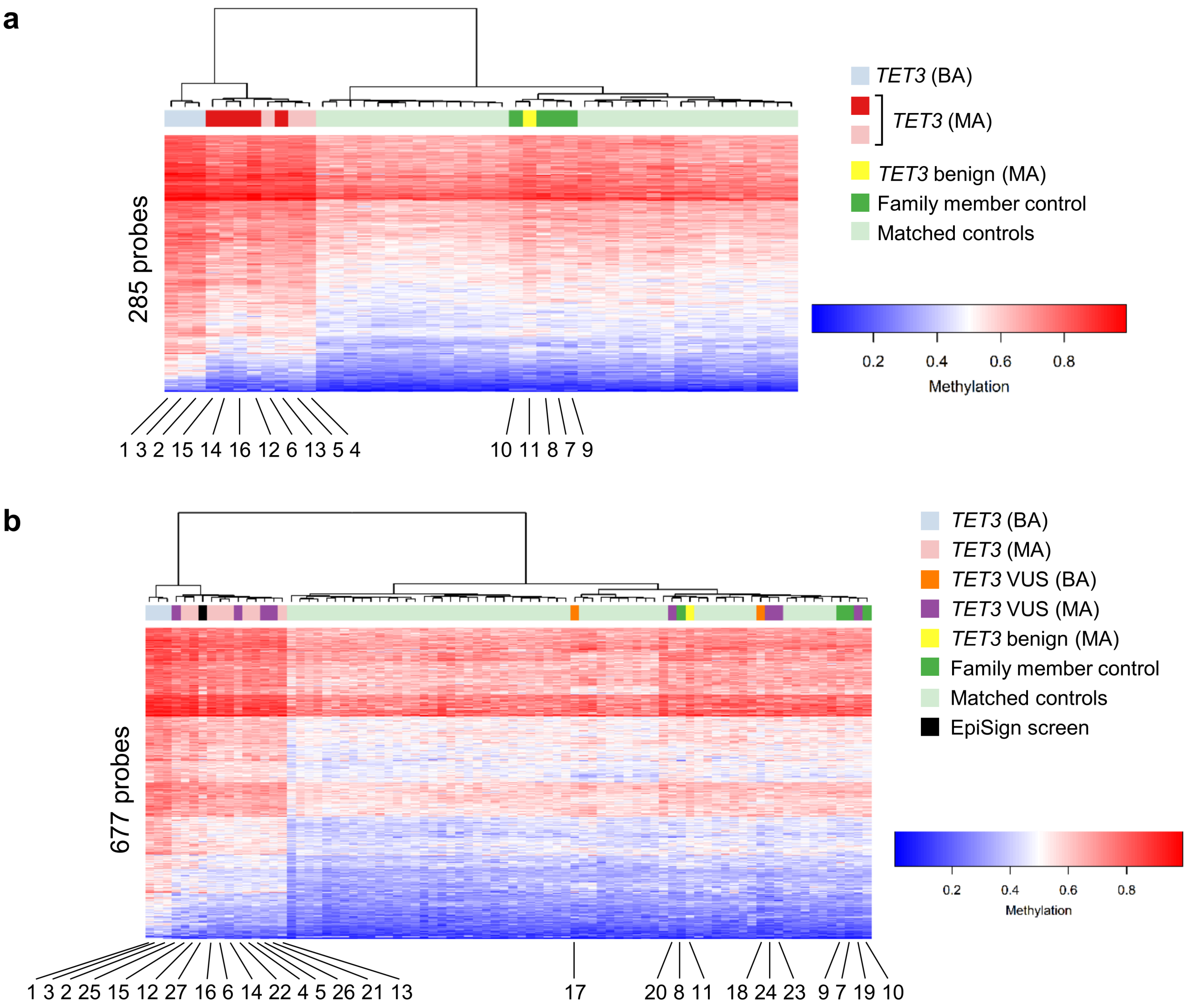

Supplementary Figure 2. Hierarchical clustering after two rounds of *TET3* episignature training. (**a**) For the first round of episignature training, the 6 pathogenic discovery samples (n=3 *TET3* (BA), light blue; n=3 *TET3* (MA), light red) and 30 matched controls (light green) were used; the validation samples were used for testing (n=5 *TET3* (MA), bright red; n=1 *TET3* benign (MA), bright yellow; n=4 family member controls, bright green). (**b**) For the second round of episignature training, the 11 *TET3* pathogenic samples in the discovery and validation cohorts (n=3 *TET3* (BA), light blue; n=8 *TET3* (MA), light red) and 55 matched controls (light green) were used for training and all of the remaining samples were used for testing (n=2 *TET3* VUS (BA), orange; n=8 *TET3* VUS (MA), purple; n=1 *TET3* benign (MA), yellow; n=1 EpiSign screen, black; n=4 family member controls, green). Each column represents one sample and each row represents one probe (CpG). The heatmap color scale from blue to red represents the DNA methylation level (beta value) from 0 (no methylation) to 1 (fully methylated). The number of probes in each episignature is indicated to the left of each heatmap. The color bar above

each heatmap uses faded colors to indicate samples used for training and vivid colors to indicate samples used for testing. Samples are color-coded according to the key in the figure and numbered corresponding to Table 1.

TET3 (BA), samples with bi-allelic pathogenic *TET3* variants; *TET3* (MA), samples with mono-allelic pathogenic *TET3* variants; *TET3* benign (MA), the benign variant that did not reduce catalytic activity *in vitro*[2]; family member controls, family members of affected individuals lacking *TET3* variants; matched controls, age-and sex-matched controls; VUS, variants of uncertain significance; *TET3* VUS (BA), samples with bi-allelic *TET3* VUS's; *TET3* VUS (MA), samples with mono-allelic *TET3* VUS's; EpiSign screen, an unknown sample identified by screening the Episign database.

Supplementary Figure 3

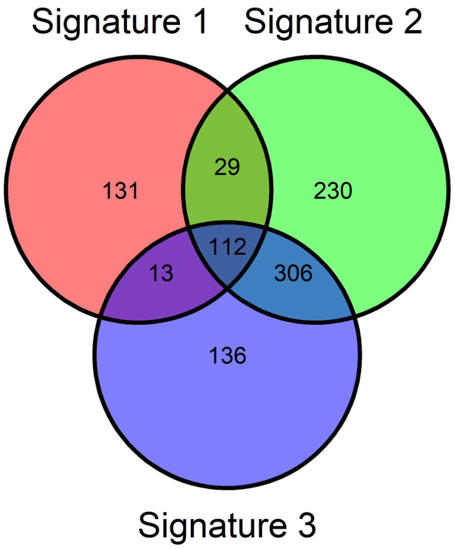

Supplementary Figure 3. Venn diagram comparing DNA methylation signatures. Episignature 1 is comprised of 285 probes, with 141 overlapping with episignature 2 and 125 overlapping with episignature 3. Episignature 2 is comprised of 677 probes, with 418 overlapping with episignature 3. Episignature 3 is comprised of 567 probes. 112 probes are common between all three episignatures.

Supplementary Figure 4

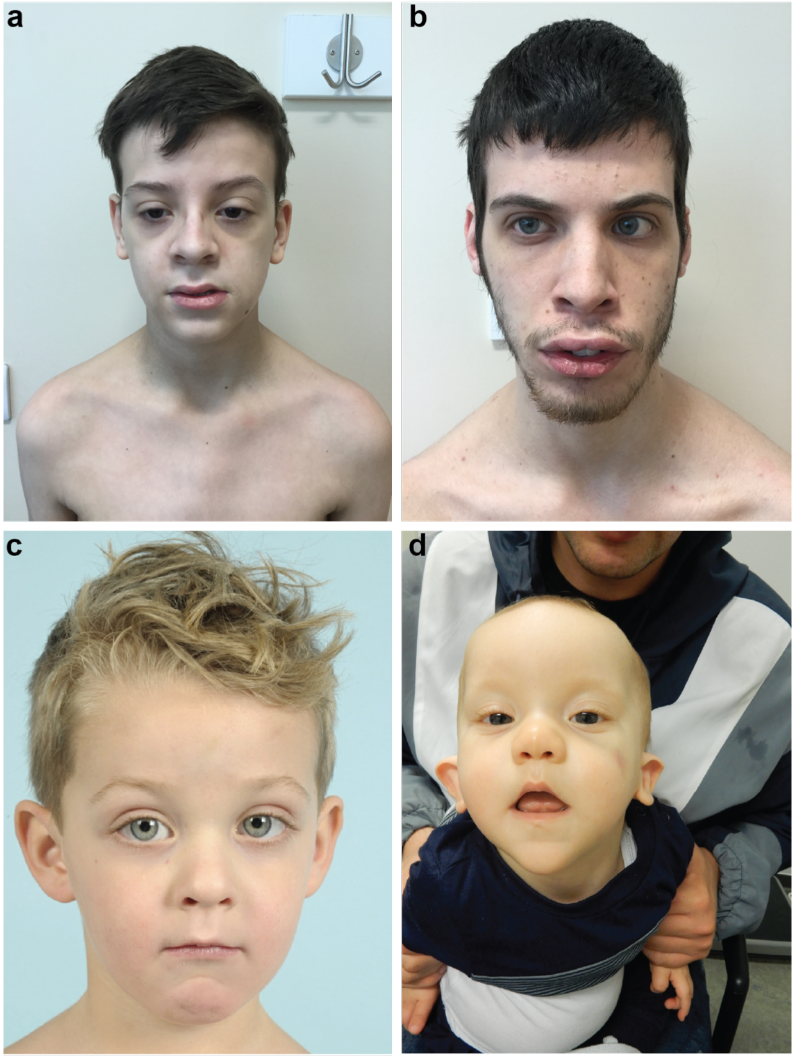

Supplementary Figure 4. Craniofacial features of individuals with BEFAHRS resulting from pathogenic variants in *TET3*. Facial features of individuals (**a**) 1-I, (**b**) 1-III, (**c**) 2, and (**d**) 5-I. Written consent was obtained for publication of photographs prior to inclusion in the study.

Supplementary Figure 5

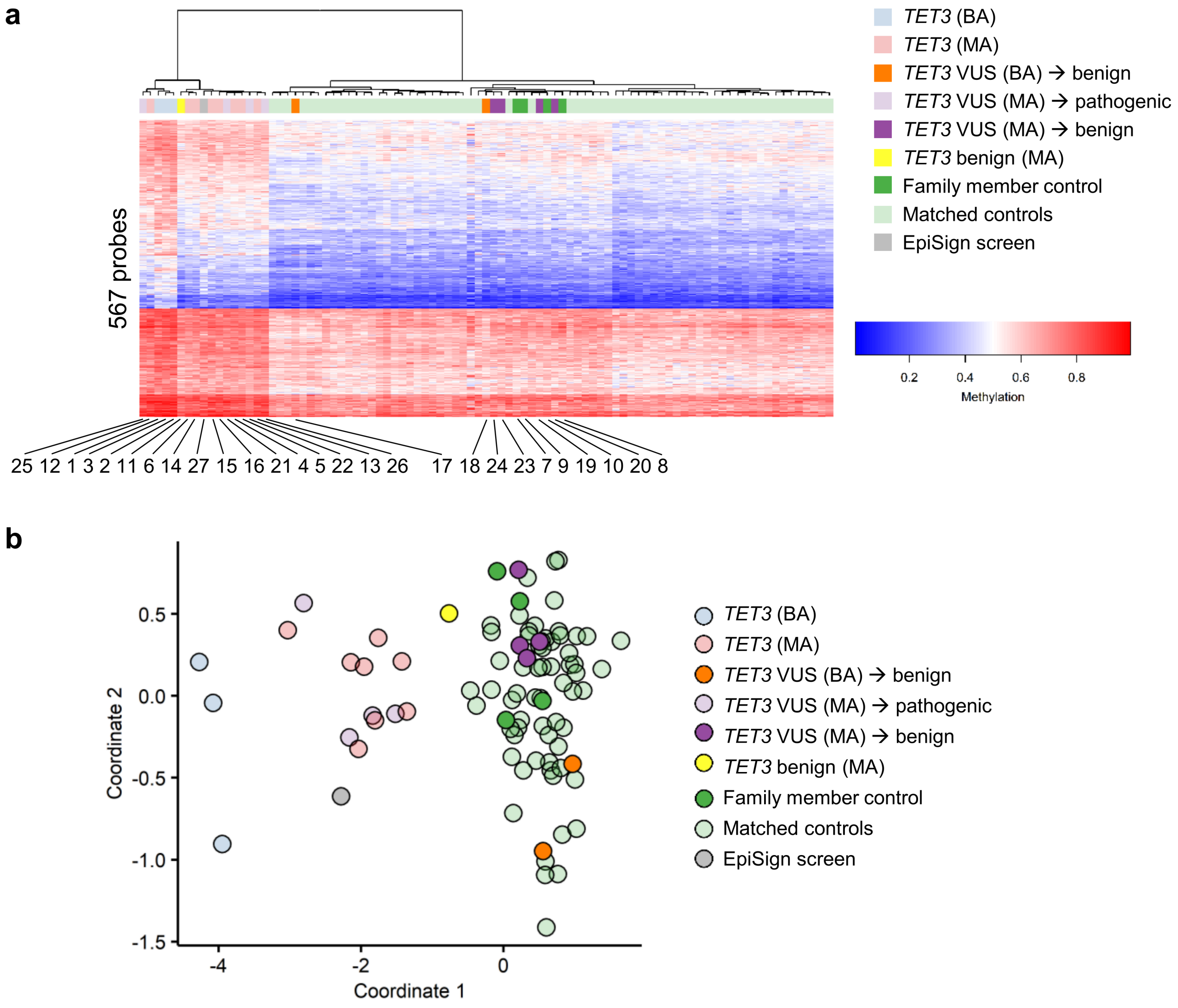

Supplementary Figure 5. Hierarchical clustering and multi-dimensional scaling after the final round of *TET3* episignature training. (**a**) Hierarchical clustering using the 16 *TET3* pathogenic samples in the discovery, validation, and testing cohorts (n=3 *TET3* (BA), light blue; n=8 *TET3* (MA), light red; n=4 *TET3* VUS (MA)→pathogenic, light purple; n=1 EpiSign screen, gray) and 64 matched controls (light green) for training and all of the remaining samples for testing (n=2 *TET3* VUS (BA)→benign, orange; n=4 *TET3* VUS (MA)→benign, purple; n=1 *TET3* benign (MA), yellow; n=4 family member controls, green). Each column represents one sample and each row represents one probe (CpG). The number of probes in the episignature is indicated on the left of the heatmap. The heatmap color scale from blue to red represents the DNA methylation level (beta value) from 0 (no methylation) to 1 (fully methylated). The color bar above the heatmap uses faded colors to indicate samples used for training and vivid colors to indicate samples used for testing. Samples are numbered

corresponding to Table 1. (**b**) Multi-dimensional scaling (MDS) plot of the same samples as in (**a**). MDS was performed by scaling of the pair-wise Euclidean distances between samples. Samples are color-coded as shown.

TET3 (BA), samples with bi-allelic pathogenic *TET3* variants; *TET3* (MA), samples with mono-allelic pathogenic *TET3* variants; *TET3* benign (MA), the benign variant that did not reduce catalytic activity *in vitro*[2]; family member controls, family members of affected individuals lacking *TET3* variants; matched controls, age-and sex-matched controls; EpiSign screen, an unknown sample identified by screening the Episign database; VUS, variants of uncertain significance; *TET3* VUS (BA)→benign, samples with bi-allelic *TET3* VUS's reclassified as benign; *TET3* VUS (MA)→benign, samples with mono-allelic *TET3* VUS's reclassified as benign; *TET3* VUS (MA)→pathogenic, samples with mono-allelic *TET3* VUS's reclassified as pathogenic.

Supplementary Figure 6

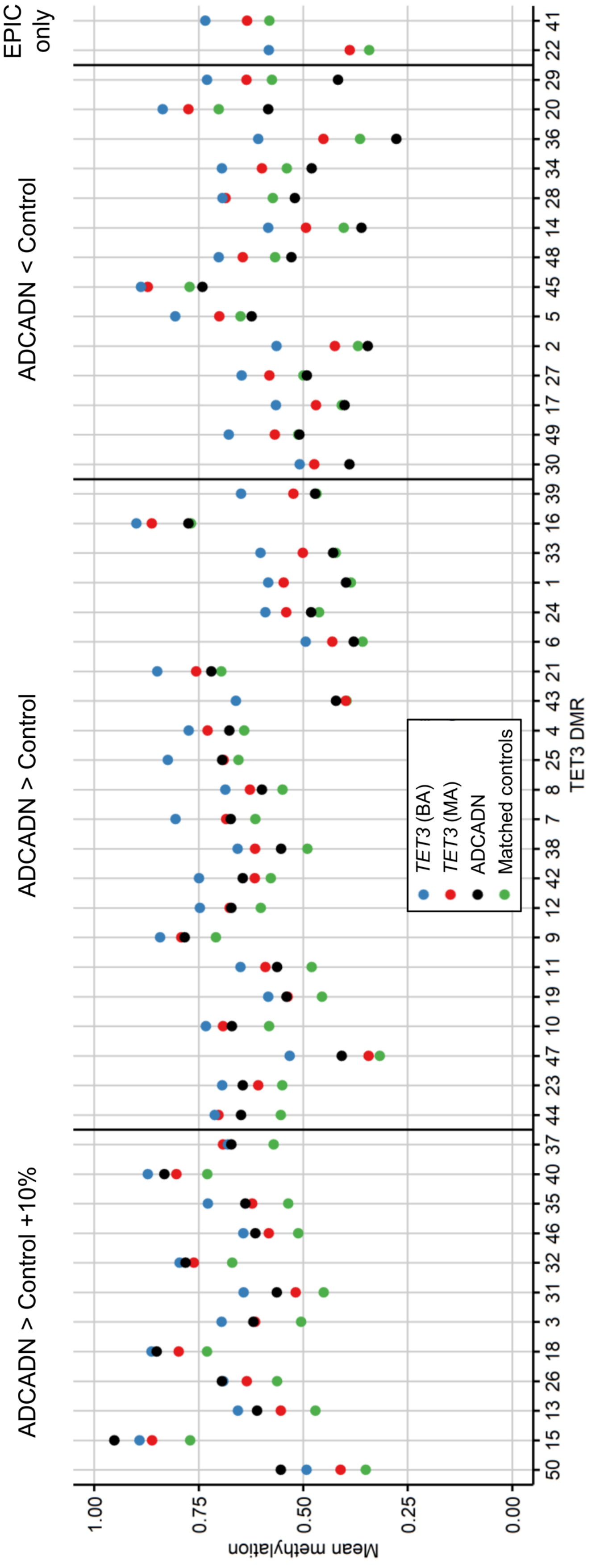

Supplementary Figure 6. Comparison of mean DNA methylation between *TET3*, *ADCADN*, and control samples at the 50 significant *TET3* DMRs. Comparison of mean methylation between ADCADN samples (n=5, black), Signature discovery samples (*TET3* (MA), n=3, red; *TET3* (BA), n=3, blue; and matched controls, n=30, green) at each of the 50 significant *TET3* DMRs. At all DMRs, *TET3* mean methylation is at least 10% greater than control methylation. The DMRs are sorted based on the methylation difference between ADCADN and control samples, with DMRs on the left passing the 10% cut-off for ADCADN samples. *TET3* DMRs 22 and 41 only have probes on the EPIC array therefore no ADCADN data (450k array) is available. ADCADN, Autosomal dominant cerebellar ataxia, deafness, and narcolepsy syndrome; *TET3* (BA), samples with bi-allelic pathogenic *TET3* variants; *TET3* (MA), samples with mono-allelic pathogenic *TET3* variants; matched controls, age-and sex-matched controls.

Supplementary Note. Case reports of individuals and families with BEFAHRS.

Family 1

Individual 1-I (**Supplementary Data 2**; proband) was first evaluated at 4 years due global developmental delay, autistic traits, pigmentary abnormalities in Blashcko's lines and petaloid hypopigmentation on calves. He was born at term (41 weeks 4 days gestation) with a birth weight of 3835 grams (62nd centile). He sat at 8 months and walked at 18 months. At 8 years, he was able to make 2-3 word sentences and count to 20. He started learning to write his name at about 8 years. He has hypermetropia and mild conductive hearing loss. Although no formal diagnosis of autism spectrum disorder (ASD), he has autistic traits, including poor social interactions with other children and multiple phobias and obsessions. He has significant intellectual disability and attended a special needs' school. At 5 years, he had a prolonged afebrile generalized tonic-clonic seizure and three additional seizures over a period of 12 months. EEG showed occasional sharp and slow wave discharges in the right central area. Brain MRI showed abnormality of the periventricular white matter. He had a murmur as a baby and echocardiogram at that time showed tiny, hemodynamically-insignificant arterial collateral from the descending aorta. He has dental enamel hypoplasia and a peripheral giant cell granuloma of the gum removed at 12 years. Examination at 12 years 11 months revealed height at the 33rd centile, weight at the 22nd centile and OFC at the 13th centile. He has tall broad forehead and a long face with deep philtrum. He has a hairless patch on scalp and skin pigmentary abnormalities. He does not have any nail changes. He has asymmetrical right pectus deformity. He has hypermobile fingers and single palmar crease on the left. He had a normal chromosome microarray analysis. Proband-only exome sequencing was performed as part of the Deciphering Developmental Disorders study, which revealed a rare variant in *TET3* (c.2036dupC; p.Thr680Tyrfs*26).

Individual 1-II is the mother of Individual 1-I. Parental testing of Individual 1-II (**Supplementary Data 2**) identified the same *TET3* variant. Initially, she was reported to be unaffected. However, evaluation at 54 years 4 months revealed a phenotype consistent with BEFAHRS. She was born at term with a birth weight of 3295 grams (41st centile). She has intellectual disability and attended a special needs school. She is able to read and write. She had behavioral problems and was short tempered as a child. She does not have a formal diagnosis of ASD. She has anxiety and depression diagnosed in adulthood. She has hypermetropia and normal vision. She has significant migraines requiring therapy and her brain MRI showed white matter changes. She also has a tall broad forehead with a long face.

Individual 1-III is the brother of Individual 1-I and the son of Individual 1-II (**Supplementary Data 2**). He was evaluated at 21 years and 1 month following his brother's genetic finding. He was born at term with a birth weight of 3977 grams (81st centile). He walked at 18m and started speaking a few words at 3-4y.

He has normal vision. He had mild left sided conductive hearing loss as a child. He has significant intellectual disability. He attended a mainstream primary school and a special needs' secondary school. He held supported jobs but had to resign due to anxiety. He was diagnosed with ASD at 11 years. He also has tremors. At 7 years, he had generalized tonic-clonic seizures and EEG showed occasional sharp and slow wave discharge in the right central area. He has not had any seizures in the last 2 years. His brain MRI is unavailable. Examination at 21 years 1 month revealed height at the 84th centile, weight at the 10th centile and OFC at the 80th centile. He has tall broad forehead with a long face and a high arched palate. He has kyphosis and hypermobile finger joints. He has striae and acne on his back. Fragile X testing was normal. Targeted testing using Sanger sequencing identified the same *TET3* variant identified in his brother (Individual 1-I) and mother (Individual 1-II).

Family 2

Individual 2 (**Supplementary Data 2**) was born at term (41 weeks 5 days), with good Apgar scores. Birthweight was 3780 grams (0 SD). His parents are non-consanguineous and of Dutch descent. No abnormalities were noted in his development during infancy. In retrospect, he had excessive drooling and sweating. At 1.5 years of age, he did not speak any words. Speech therapy was started at age 2 years. Eustachian tube placement did not improve speech. Motor delay was noted at 3 years of age. He was toilet trained just before 4 years of age. At the age of 4 years his active speech was still limited and he was very difficult to understand, especially to non-family members. He continued to have excessive drooling. Non-verbal cognitive testing (SON-R 2.5-7) at 4 years of age showed a discrepancy between performance scores (59) versus reasoning (93). He had a significant motor delay on the Movement-ABC (0.1 percentile) which persisted after 7 months of physical therapy. Around age 4-5 he repeatedly had throat infections leading to adenoidectomy and tonsillectomy. He is a sweet boy who is shy and insecure due to his awareness of his disability. He needs a safe environment, individual attention and lots of repetition to achieve active learning. He learned to write his first letters at 6 years of age, and at age 8, is now able to read. He is 6-12 months behind in school. He has myopia (-3.5 diopters). Physical examination at age 5 years, 11 months showed a friendly boy, with dysarthria. Height 118.4 cm (-0.1 SD), weight 24 kg (weight to height +1.4 SD), OFC 52.5 cm (+0.4 SD). He has a long, oval-shaped, and hypotonic face; a tall and broad forehead, a frontal upsweep, arched eyebrows, prominent eyes, a thin upper lip, clinodactyly of digits IV and V of the feet. Moist hands and feet were noted. Chromosomal microarray, Fragile X testing, and metabolic testing in plasma and urine showed no abnormalities. He was found on trio exome sequencing to have a variant in *TET3* (c.3100C>T; p.Arg1034*).

Family 3

Individual 3 (**Supplementary Data 2**) is a 21-year-old man, the second of four children from non-consanguineous Caucasian parents, born at term after an uneventful pregnancy. Family history is negative for psychiatric problems. His early development was largely normal, though he was a bit clumsy with borderline gross motor delays requiring physical therapy and had some fine motor difficulties involving writing, for which he received occupational therapy. He did have problems in social interaction, but with some extra support he finished primary school with good grades and started at a regular secondary school. Hearing and vison were normal, and he was generally healthy. At 14 years of age, he started to exhibit difficult behaviors requiring a special needs school for children with behavioral problems. He developed psychiatric problems with severe anxiety, depression, panic attacks, concentration problems, psychotic periods with aggression, hallucinations, and self-mutilation. In between he has periods of normal behavior, during which he is friendly, cooperative, and exhibits good verbal expression. At the age of 19 years, his total IQ was 71, with a disharmonic profile (verbal IQ 83, performance IQ 62). These results may be influenced by his psychiatric features. Genetics work up revealed normal metabolic screening and array CGH with a paternally-inherited deletion at 10q23.1. Trio exome sequencing revealed a heterozygous *de novo* variant in *TET3* (c.2732G>A; p.Arg911Gln). Currently, he lives in a residential setting.

Family 4

Individual 4 (**Supplementary Data 2**) was born at 41 weeks and 5 days gestation via vacuum-assisted vaginal delivery after an uncomplicated pregnancy. His birth weight was 3570 grams. He had infantile hypotonia and walked at 15-16 months of age. He required physical and occupational therapy due to gross and fine motor delays. His speech development was delayed as well, and he began to speak at 2.5-3 years of age and required speech therapy. He goes to a special school and has an IQ of about 60. Evaluations for attention deficit hyperactivity disorder (ADHD) and autism spectrum disorder were negative, but he is sensitive to sounds and other stimulation. He had chronic otitis media with associated intermittent hearing loss and required three sets of tympanostomy tubes. He also required tonsillectomy, but there were no concerns for other recurrent infections. His vision, sleep, and diet are normal. He has had a single foot fracture. His younger brother has more mildly delayed development and attends a regular school. His paternal half-sister had an IQ of 80-90 and attended a special school. A maternal uncle went to a special school. Physical examination of the proband revealed the following growth parameters: length +0.44 SD (target height -0.41SD), weight -0.03SD, and head circumference +0.51 SD on local growth chart. He had a long face, arched eyebrows, hypotelorism, somewhat low-set ears, and an open mouth appearance/hypotonic face. The following genetics work up was normal and did not reveal a cause for his clinical findings: metabolic testing of blood and urine, SNP array, Fragile X testing, and myotonic dystrophy type 1 (DMPK gene) testing. However, trio exome sequencing revealed a *de novo*, heterozygous variant in *TET3* (c.5048G>A; p.Arg1683His).

Family 5

Individual 5-I (**Supplementary Data 2**) was diagnosed prenatally with Tetralogy of Fallot. Prenatal microarray was normal and parents declined prenatal exome sequencing. After birth, the clinical geneticist noticed triangulated CHARGE-like ears and triangular nails. Screening for clinical findings of CHARGE syndrome did not find any additional abnormalities. Trio exome sequencing was performed (prior to the delineation of *TET3* deficiency) and did not yield a causative variant. At 6 months, shortly after his cardiac surgery, his mother became worried that he could have Kabuki syndrome. The geneticist thought this might be possible given his facial appearance and protruding ears and requested an Episign DNA methylation array test to exclude Kabuki syndrome. This test came back negative for known conditions. After several months, a *TET3*-deficient signature was found and was reported back to the geneticist. After requesting re-analysis of the trio exome sequencing, specifically to evaluate for variants in *TET3*, a maternally-inherited nonsense variant (c.738 C>A; p.Cys246*) was identified. Of note, in the mother (Individual 5-II; **Supplementary Data 2**), 125 sequence reads showed the variant, and 160 reads showed the reference allele (p=0.022 for a 50% distribution, which would be expected for a full heterozygous variant), and so it is possible that she is mosaic for the variant. The proband (Individual 5-I; **Supplementary Data 2**) has 149 sequence reads showing the variant and 154 reads showing the reference allele. The parents were not available for further testing via Episign at the time of submission. The mother (Individual 5-II; **Supplementary Data 2**) had difficulty with social interactions in childhood but completed primary and secondary school, as well as two years of cook training at a lower level. The proband (Individual 5-I; **Supplementary Data 2**) crawled around 12 months of age. At 14 months, he spoke several words (mum/dad/food), had begun to cruise but was not walking independently, and his height and weight were less than two standard deviations below the mean for age. He was found to have low IGF-1 and IGF-BP3. Of note, it remains unclear whether the Tetralogy of Fallot is related to the variant in *TET3*.

Supplementary References

1. Cao, J. *et al.* A human cell atlas of fetal gene expression. *Science* **370**, doi:10.1126/science.aba7721 (2020).
2. Beck, D. B. *et al.* Delineation of a Human Mendelian Disorder of the DNA Demethylation Machinery: TET3 Deficiency. *American journal of human genetics* **106**, 234-245, doi:10.1016/j.ajhg.2019.12.007 (2020).

Supplementary Data 1. Differentially methylated regions in *TET3*-deficient samples

Supplementary Data 2. Clinical features of BEFAHRS
