# Supplementary material for: Deficiency of TET3 leads to a genome-wide DNA hypermethylation episignature in human whole blood
Source: NPJ Genom Med. 2021 Nov 8;6:92. doi: 10.1038/s41525-021-00256-y (PMC8576018; doi:10.1038/s41525-021-00256-y)
Supplement: Supplementary file 4 — Reporting Summary [file 41525_2021_256_MOESM4_ESM.pdf]

nature research

Corresponding author(s): Jill Fahrner and Bekim Sadikovic

Last updated by author(s): Apr 22, 2021

Reporting Summary

Nature Research wishes to improve the reproducibility of the work that we publish. This form provides structure for consistency and transparency in reporting. For further information on Nature Research policies, see our Editorial Policies and the Editorial Policy Checklist.

Statistics

For all statistical analyses, confirm that the following items are present in the figure legend, table legend, main text, or Methods section.

n/a	Confirmed	
☐	☒	The exact sample size (*n*) for each experimental group/condition, given as a discrete number and unit of measurement
☐	☒	A statement on whether measurements were taken from distinct samples or whether the same sample was measured repeatedly
☐	☒	The statistical test(s) used AND whether they are one- or two-sided<br>*Only common tests should be described solely by name; describe more complex techniques in the Methods section.*
☐	☒	A description of all covariates tested
☐	☒	A description of any assumptions or corrections, such as tests of normality and adjustment for multiple comparisons
☐	☒	A full description of the statistical parameters including central tendency (e.g. means) or other basic estimates (e.g. regression coefficient) AND variation (e.g. standard deviation) or associated estimates of uncertainty (e.g. confidence intervals)
☐	☒	For null hypothesis testing, the test statistic (e.g. *F*, *t*, *r*) with confidence intervals, effect sizes, degrees of freedom and *P* value noted<br>*Give P values as exact values whenever suitable.*
☒	☐	For Bayesian analysis, information on the choice of priors and Markov chain Monte Carlo settings
☒	☐	For hierarchical and complex designs, identification of the appropriate level for tests and full reporting of outcomes
☒	☐	Estimates of effect sizes (e.g. Cohen's *d*, Pearson's *r*), indicating how they were calculated

Our web collection on statistics for biologists contains articles on many of the points above.

Software and code

Policy information about availability of computer code

Data collection	Data was collected by scanning Illumina microarrays using Illumina's commercial software: iScan Control Software version 3.3.28.
Data analysis	Analysis was done using open source R packages as specified in the text. Packages and version numbers were: R 3.6.2, minfi 1.32.0, MatchIt 3.0.2, limma 3.42.2, gplots_3.0.4, e1071 1.7-3, DMRcate 2.0.7. Version numbers are added to the methods section in revised text.

For manuscripts utilizing custom algorithms or software that are central to the research but not yet described in published literature, software must be made available to editors and reviewers. We strongly encourage code deposition in a community repository (e.g. GitHub). See the Nature Research guidelines for submitting code & software for further information.

Data

Policy information about availability of data

All manuscripts must include a data availability statement. This statement should provide the following information, where applicable:

- Accession codes, unique identifiers, or web links for publicly available datasets
- A list of figures that have associated raw data
- A description of any restrictions on data availability

The summarized, anonymized data for each subject is described in the study. The raw DNA methylation data are not available due to the institutional and ethics restrictions. Software used in this study is publicly available and detailed analytical methodology is as previously reported (Ref 9; Aref-Eshghi et al., AJHG 2020).

Field-specific reporting

Please select the one below that is the best fit for your research. If you are not sure, read the appropriate sections before making your selection.

☒ Life sciences ☐ Behavioural & social sciences ☐ Ecological, evolutionary & environmental sciences

For a reference copy of the document with all sections, see nature.com/documents/nr-reporting-summary-flat.pdf

Life sciences study design

All studies must disclose on these points even when the disclosure is negative.

Sample size	An inherent limitation in studying rare syndromes is small sample sizes. The performance of the initial “signature discovery” classifier is demonstrated empirically by its ability to correctly classify the “signature validation” samples and by the correlation between the Support Vector Machine (SVM) results and unsupervised clustering results. One of the goals of the multiple rounds of episignature discovery performed in the study was to identify additional samples that can be added to the episignature to increase sample size and robustness. The final classifier uses 16 samples. Data analysis and sample size are similar to ones described in the majority of other genetic disorders with identified episignatures.
Data exclusions	No data were excluded.
Replication	For identification of the episignature, multiple rounds of episignature discovery identifying overlapping probes and classifying samples into similar groups showed the data could be replicated. For the comparison of DMR-associated gene expression to the expression of other genes in neurons, the analysis was performed in two slightly different ways and similar results were obtained, confirming the data could be replicated. Please see Methods section for additional details.
Randomization	Samples were allocated into experimental groups (case samples, control samples, and other syndrome samples including individuals with variants of uncertain significance) based on genetic and clinical assessments as described in the text. Covariates of patient age and sex were controlled for by matching case and controls samples by age and sex as described in the text. Covariate of blood cell composition was controlled for by including estimated blood cell proportions in the regression modeling as described in the text.
Blinding	Investigators were not blinded to group allocations for majority of the analysis since the process of determining group allocation was the objective of the study. Samples were assigned to groups (for example case/pathogenic or control/benign) at several steps during the analysis as described in the text. Investigators were blinded in part of the analysis described as “EpiSign Screen” where sample was identified as a case/pathogenic sample where presence of a pathogenic variant was confirmed after the initial classification.

Reporting for specific materials, systems and methods

We require information from authors about some types of materials, experimental systems and methods used in many studies. Here, indicate whether each material, system or method listed is relevant to your study. If you are not sure if a list item applies to your research, read the appropriate section before selecting a response.

Materials & experimental systems

n/a	Involved in the study	
☒	☐	Antibodies
☒	☐	Eukaryotic cell lines
☒	☐	Palaeontology and archaeology
☒	☐	Animals and other organisms
☐	☒	Human research participants
☐	☒	Clinical data
☒	☐	Dual use research of concern

Methods

n/a	Involved in the study	
☒	☐	ChIP-seq
☒	☐	Flow cytometry
☒	☐	MRI-based neuroimaging

Human research participants

Policy information about studies involving human research participants

Population characteristics	THis is a newly-described and ultra-rare disorder. We collected all interested participants with TET3 variants, multiple age- and sex-matched controls, and family member controls. Males and females were included. Age ranged from 1 to 64 years. Genotypes of all participants and additional details are in Table 1.
Recruitment	THis is a newly-described and ultra-rare disorder. Clinicians and researchers with patients interested in research and who had variants in TET3 reached out to the corresponding authors via GeneMatcher or directly via email. The study was explained to the researchers and participants, who were properly consented and enrolled.
Ethics oversight	Johns Hopkins Institutional Review Board (IRB) and Western University Research Ethics Board, as well as the ethics boards

Ethics oversight	and IRBs of other co-authors' institutions.

Note that full information on the approval of the study protocol must also be provided in the manuscript.

Clinical data

Policy information about clinical studies

All manuscripts should comply with the ICMJE guidelines for publication of clinical research and a completed CONSORT checklist must be included with all submissions.

Clinical trial registration	This is not a clinical trial.
Study protocol	This is not a clinical trial.
Data collection	This is not a clinical trial.
Outcomes	This is not a clinical trial.
